# KMT2D regulates activation, localization, and integrin expression by T-cells

**DOI:** 10.3389/fimmu.2024.1341745

**Published:** 2024-05-03

**Authors:** Sarah J. Potter, Li Zhang, Michael Kotliar, Yuehong Wu, Caitlin Schafer, Kurtis Stefan, Leandros Boukas, Dima Qu’d, Olaf Bodamer, Brittany N. Simpson, Artem Barski, Andrew W. Lindsley, Hans T. Bjornsson

**Affiliations:** ^1^ Division of Allergy & Immunology, Cincinnati Children’s Hospital Medical Center, Cincinnati, OH, United States; ^2^ McKusick-Nathans Department of Genetics, The Johns Hopkins University School of Medicine, Baltimore, MD, United States; ^3^ Department of Biostatistics, Johns Hopkins Bloomberg School of Public Health, Baltimore, MD, United States; ^4^ Division of Human Genetics, Cincinnati Children’s Hospital Medical Center, Cincinnati, OH, United States; ^5^ Division of Genetics and Genomics, Boston Children’s Hospital, Boston, MA, United States; ^6^ The Roya Kabuki Program, Boston Children’s Hospital, Boston, MA, United States; ^7^ Division of Genetics and Genomics, Broad Institute of MIT and Harvard University, Cambridge, MA, United States; ^8^ Department of Pediatrics, University of Cincinnati College of Medicine, Cincinnati, OH, United States; ^9^ Faculty of Medicine, The University of Iceland, Reykjavik, Iceland; ^10^ Department of Genetics and Molecular Medicine, Landspitali University Hospital, Reykjavik, Iceland

**Keywords:** Kabuki syndrome (KS), KS1-associated immune deficiency (KSAID), thymocyte, recent thymic emigrant (RTE), *Itgal*, *Itgb7*, integrin switching

## Abstract

Individuals with Kabuki syndrome present with immunodeficiency; however, how pathogenic variants in the gene encoding the histone-modifying enzyme lysine methyltransferase 2D (KMT2D) lead to immune alterations remain poorly understood. Following up on our prior report of KMT2D-altered integrin expression in B-cells, we performed targeted analyses of KMT2D’s influence on integrin expression in T-cells throughout development (thymocytes through peripheral T-cells) in murine cells with constitutive- and conditional-targeted *Kmt2d* deletion. Using high-throughput RNA-sequencing and flow cytometry, we reveal decreased expression (both at the transcriptional and translational levels) of a cluster of leukocyte-specific integrins, which perturb aspects of T-cell activation, maturation, adhesion/localization, and effector function. H3K4me3 ChIP-PCR suggests that these evolutionary similar integrins are under direct control of KMT2D. KMT2D loss also alters multiple downstream programming/signaling pathways, including integrin-based localization, which can influence T-cell populations. We further demonstrated that KMT2D deficiency is associated with the accumulation of murine CD8^+^ single-positive (SP) thymocytes and shifts in both human and murine peripheral T-cell populations, including the reduction of the CD4^+^ recent thymic emigrant (RTE) population. Together, these data show that the targeted loss of *Kmt2d* in the T-cell lineage recapitulates several distinct features of Kabuki syndrome-associated immune deficiency and implicates epigenetic mechanisms in the regulation of integrin signaling.

## Introduction

Epigenetic dysregulation has emerged as a pathologic driver of many diseases, including primary immune deficiencies (1–3). Kabuki syndrome type 1 (KS1) is an autosomal dominant, congenital, epigenetic disorder commonly associated with heterozygous loss-of-function variants in the lysine methyltransferase 2D gene, *KMT2D* (also known as *MLL2*/*MLL4*/*ALR*). KS1 has a complex phenotype, including disruption of craniofacial, skeletal, cardiac, cognitive, and immune development. KS1 is frequently associated with specific forms of immune dysfunction, including humoral immunodeficiency, autoimmunity, and lymphoproliferation, which contribute to the morbidity and mortality seen in individuals with KS1 (1, 3, 4). KS1-associated immune deficiency (KSAID) can be partially explained through the known role of KMT2D in B-cell survival/cell cycle regulation, differentiation, and peripheral tissue homing (5, 6). Specifically, KMT2D influences the expression of its target gene integrin beta 7 (*ITGB7*) and the ITGA4-ITGB7 integrin heterodimer (also known as lymphocyte Peyer’s Patch adhesion molecule-1 [LPAM1]) in mesenteric lymph node–derived lymphocytes and thus disrupts Peyer’s Patch size, quantity, and function (2). However, the contribution of T-cells to KSAID pathogenesis is currently not well understood. Recent studies suggest that KMT2D insufficiency in T-cells may be disruptive as researchers have identified *KMT2D* mutations in 20% of peripheral T-cell lymphomas-not otherwise specified (PTCL NOS), a disease with notable T-cell dysfunction tied to dysregulated expression of the cell cycle and adhesion genes (7, 8). Furthermore, dysregulated cell cycle/proliferation has been described across many KMT2D-deficient models systems, including neurons and cancer cells (9, 10). However, the contribution of T-cells to the KS1 immune deficiency phenotype has remained unclear. Recent studies in *Kmt2d* knockout (KO) (of exons 16–19) mice showed multiple T-cell abnormalities including significant drops in all peripheral T-cell subsets, a reduction in thymic CD4^+^ T regulatory cells, loss of the interferon gamma (IFNγ) silencer CNS-28, and disrupted post-stimulation-induced survival of peripheral CD8^+^ T-cells (11–13). Additionally, an immune phenotyping study of individuals with KS1 suggested decreased CD4^+^ memory cells (14); however, the use of appropriate reference ranges negated this finding (15). Recently, KMT2D’s role in long-range chromatin interactions and in modulation of the chromatin structure has become a focus (11, 13). However, the contribution of KMT2D across T-cell development and how *KMT2D* T-cell-specific haploinsufficiency (KS1) impacts peripheral T lymphocyte populations driving KSAID pathogenesis remain unaddressed. Further understanding of T-cell development and function in individuals with KS1 may improve care for all patients with immune deficiency and broaden our basic understanding of epigenetic regulation of T-cell development.

Here, we show that KMT2D directly regulates integrin expression, specifically *Itgae*, *Itgal*, *Itgb2*, and *Itgb7*, throughout the T-cell lineage, thus modulating T-cell maturation, activation, and localization. Additionally, KMT2D regulates the key thymic egress licensing transcription factor *Klf2*. In the periphery, we demonstrate that *Kmt2d* deficiency leads to changes in immune populations, such as memory skewing. Together, these data demonstrate that *Kmt2d* regulates integrins through T-cell-intrinsic epigenetic mechanisms and thus is a regulator of adaptive immunity.

## Methods

### Patient consent and sample collection

The parents of the human subjects provided written consent for enrollment in this study (Cincinnati Children’s Hospital Medical Center [CCHMC] Institutional Review Board [IRB], protocol #2012-4636 and/or protocol #2020-0570). All KS1 cohort peripheral blood lymphocyte subpopulation analyses were performed in the CCHMC Diagnostic Immune Laboratory, and the collected data were compiled from a review of medical records. All clinical evaluations were performed at Clinical Laboratory Improvement Amendments (CLIA)–certified clinical laboratories and reported with the age-matched normal ranges. All 16 KS1 cohort patients underwent *KMT2D* sequencing (reference transcript: NM_003482). KMT2D domains were mapped according to UniProt Database (O14686). KMT2D mutations and type (frameshift, nonsense, missense, splice variants) associated with the individual’s recorded DNA mutation were generated *via* VarSome (https://varsome.com/) (16) for the Lolliplot (https://www.cbioportal.org/mutation_mapper) (17, 18). Lolliplots were manually altered in order to include additional variant information through color and shape. Values found within (closed) and outside (open) the reference range (2.5 – 97.5 percentile) are both displayed on the plots. The significance was determined using the binomial test, with the null hypothesis being that individuals with KS1 have the same probability of falling outside the reference range (2.5 - 97.5 percentile) equal to 5%, that is, the same as healthy age-matched individuals, and the *P*-value generated is listed on the graphs in **Figure 6**. The lower limit of normal (LLN) for assessment of cardiac surgery is calculated using the KS1 value divided by the lowest value (2.5 confidence value) of the reference range.

### Mice

Mice were housed in accordance with NIH guidelines, and experimental protocols were approved by the Institutional Animal Care and Use Committee of CCHMC and Johns Hopkins University. Experimental observations of phenotypes were replicable at both institutions. The thymus, spleen, and/or blood were collected for flow cytometry and phenotyping analysis. Magnetic bead sorting was performed to isolate single-positive (SP) thymocytes or peripheral T-cell subsets for assessment of Cre recombinase efficiency and for RNA isolation, RNA-seq library preparation and sequencing. *Kmt2d^+/βgeo^* (also known as *Mll2Gt^(RRt024)Byg^*) mice (gene trap) were obtained from Bay Genomics (Berkeley, CA) and contain a powerful splice enhancer integrated in between exons prior to the SET domain. These mice show many features in common with patients with KS (19). *Kmt2d^+/βgeo^* animals were backcrossed onto a pure C57BL/6 background (verified by SNP array *via* Taconic Labs) and are maintained through breeding heterozygous carriers with fresh C57BL/6J (B6; Jackson Laboratories) as homozygous *Kmt2d^βgeo/βgeo^* mice are embryonic lethal.

*Kmt2d* ^-SET-fl/fl^ (also known as *Mll4* ^-SET-fl/fl^ mice), a kind gift from Dr. Kai Ge (NIH) that have been previously described (20, 21), were crossed with Cre recombinase (Cre) under various promoters. This mouse strain is not the same as the publicly available *Kmt2d* ^tm1.1Kaig/J^ (also known as *Mll4* ^fl/fl^) mouse from Jackson Laboratories (Strain #032152), which we refer to in this document (referencing some prior *Kmt2d* literature (11–13) and scRNA analyses [GSE217656] as *Kmt2d* ^-exon16-19-fl/fl^ to differentiate it from *Kmt2d* ^-SET-fl/fl^. Briefly, *Kmt2d* ^-SET-fl/fl^ mice harbor LoxP sites around *Kmt2d* exons 50 and 51 and disrupt the SET domain through a frameshift causing a stop codon at exon 52 upon exposure to Cre, which leads to a truncated protein (276 amino acid truncation at the carboxy-terminal end).

Conditional mice will sustain *Kmt2d* loss throughout thymocyte development and into the mature T lymphocytes in the periphery, thus allowing us to determine the role of *Kmt2d* in T-cell development. *Vav1*-iCre (a kind gift from Senad Dianovic, CCHMC, but can also be purchased from The Jackson Laboratory, Bar Harbor, USA; Strain #008610) turns on at the hematopoietic stem cell stage, whereas the two additional models used to conditionally delete T-cells, *Lck*-Cre^Mar^ (The Jackson laboratory; Stain #003802) reportedly turns on around DN3 (22) and *CD4*-Cre (a kind gift from David Hildeman, CCHMC but can also be purchased from The Jackson laboratory; Strain #022071) turns on around DN4 (22) (see **Supplementary Figure 1**).

All mice used in these studies were adults (ranging 1 – 6 months old), with the majority of mice 2 – 3 months of age. All mutant mice used were evaluated against their same-sex littermate controls for age-/sex-related normalizing purposes. Mice were sacrificed *via* intraperitoneal injection of sodium pentobarbital (65 mg mL^−1^; 200 μL – 300 μL) after which the thymus, spleen, mesenteric lymph node, and/or blood were collected. Using a 3 mL syringe plunger (#309656; Becton Dickinson [BD], Franklin Lakes, USA), the organs were mashed through a T-cup strainer (#352350; Corning Inc., Glendale, USA) using 5 – 10 mL of 0.5% DNase I (DN25-1G; Sigma, St. Louis, USA) in complete RPMI 1640 (cRPMI; RPMI 1640 [#10-040-CV; Corning/Mediatech Inc., Manassas, USA] containing 10% heat inactivated fetal bovine serum [#16140-071; Invitrogen]). Peripheral blood was collected from live mice for analysis *via* submandibular phlebotomy into K_2_EDTA tubes (Microtainer, #365974; BD). Cells were collected and spun down at 500 g × 5 min. The 1 million cells collected from tissues or blood were strained and used for flow cytometric analyses or for recent thymic emigrant T-cell receptor excision circles assessment.

### Flow cytometry analysis

Cells were spun down at 500 g for 5 min to pellet and resuspended in 5 mL of cRPMI. Cells were stained using a dilution of 1:400 of anti-mouse CD16/CD32 (Fc Shield, #70-0161-U100; Tonbo Biosciences, San Diego, USA) in cRPMI for 15 min, washed with cRPMI, spun down, and incubated with the following antibodies in cRPMI at 1:200 for 30 min: CD4-BV650 (#100469; BioLegend, San Diego, USA), CD8-BV605 (#100744; BioLegend), CD25-PE-Cy7 (#102016; BioLegend), CD44-AF488 (#103016; BioLegend), and additional panel antibodies (TCRβ, ITGAL, ITGAE, MHCI, CD69, CCR7, CD24) or the peripheral (splenic) antibody panel; see **Supplementary Table 1** and **Supplementary Figure 2** for antibody information and gating strategies, respectively. For analysis only, cells were washed with 1× DPBS (10× DPBS diluted with Millipore water 1:10; #14200-075; Thermo Fisher Scientific) twice then stained at 1:1,000 with Zombie UV Fixable Live/Dead (blue-fluorescent reactive dye #L23105A; Thermo Fisher Scientific) for 15 min at room temperature. Cells were washed, spun down, and fixed with either 4% diluted paraformaldehyde (#15710-S; Electron Microscopy Sciences, Hatfield, USA) or with Cytofix/Cytoperm (#554722; BD) for 20 – 30 min, washed with 1× DPBS, spun down, and stored in 1× DPBS or cRPMI until run on the LSRFortessa I flow cytometer (BD, maintained/run at CCHMC) or FACSVerse (BD, run at JHU). Flow cytometry files were analyzed using FlowJo (TreeStar LLC, now BD, Ashland USA).

### Magnetic bead sorting

Cells were spun down at 500 *g* for 5 min to pellet and resuspended in 5 mL of cRPMI. Single cells were depleted of both CD4^+^ double-positive (DP) and CD4^+^ SP cells using a CD4^+^ (L3T4) positive selection kit (Microbeads; #130-117-043; Miltenyi Biotec, Gaithersburg, USA). The resulting flow-through contains both double-negative [DN] and CD8^+^SP cells. Next, the CD8a^+^ (Ly-2) positive selection kit was applied to the DN/CD8^+^SP containing flow-through to isolate CD8^+^SP thymocytes per manufacturer’s instructions (Microbeads, #130-117-044; Miltenyi Biotec). The second flow-through resulted in the DN population, whereas the magnetic elute resulted in the CD8^+^SP population. Conversely, CD4^+^SP isolation was used in the opposite microbead sequence (CD8 kit then CD4 kit). Briefly, both these kits used a 10 min incubation with either CD4^+^- or CD8^+^-specific microbeads followed by applying cells to the magnet-associated column, thereby capturing the CD4^+^ or CD8^+^ cells, relative to the antibody-microbead used. CD4^+^ and CD8^+^ peripheral T-cells require only one positive microbead kit for isolation.

### Assessment of Cre recombination efficiency

Recombination of magnetic sorted thymocyte and peripheral cells (pool from the spleen and lymph nodes) and CD4^+^ and CD8^+^ cells (see **Supplementary Figure 1** for Cre recombination activity documented in prior literature [**Supplementary Figure 1A**] and location of our designed primers and predicted outcomes [**Supplementary Figure 1B**; **Supplementary Table 2**]) was assessed by determining the expression or loss of the exon 50/SET domain through genomic DNA quantitative polymerase chain reaction (PCR). CD4^+^ and CD8^+^ cells were sorted through magnetic bead separation (see Magnetic bead sorting procedure above) and genomic DNA was isolated *via* a QIAamp DNA Micro Kit (#56304; Qiagen LLC, Germantown, USA) per manufacturers’ directions and quantified optically by Qubit 2.0 Fluorometer (#Q32866; Thermo Fisher Scientific) using Qubit dsDNA HS Reagent/Buffer (#Q32854A/Q32854; Thermo Fisher Scientific). A quantitative PCR reaction was performed using 10 ng (in 3 µL volume) of extracted DNA per reaction, 2 µL of combined 1 µM forward and reverse primers (see **Supplementary Table 2**), and 5 µL SYBR Green Master Mix (#A25742; Applied Biosystems, Waltham, USA) and run on the QuantStudio 7 Flex (Applied Biosystems) or ABI ViiA7 (Applied Biosystems) in a MicroAmp Optical 384-well reaction plate (#A4309849; Applied Biosystems). The CT values were then placed in the following equation: 2^-(Mutant [CT of^ *^Kmt2d^* ^-Exon50/CT of^ *^Kmt2d^* ^-Total R20] – Control [CT of^ *^Kmt2d^* ^-Exon50/CT of^ *^Kmt2d^* ^-Total R20])^ for recombination assessment.

### Assessment of migration

Choice of the Transwell pore size (3 µM compared with 5 µM) is informative about types of thymocyte migration (for example: 3 µM requires cellular contractility [using Myosin IIa] and polarization/shape changing [*Mst1* pathway] (23, 24) compared with 5 μM pores are general un-impeded migration). As previously described, control and *Kmt2d^+/βgeo^* bulk thymocytes were either pretreated with LFA1 blocking antibody (Clone M17/4, #BE0006; Bio X Cell) at 100 μg mL^−1^ or left untreated for 30 min, then washed and loaded at 5 × 10^6^ bulk thymocytes per 100 μL of PBS containing BSA on the top of a 24-well (6.5 mm) 3 μM pore (#3415; Corning) or 5 μM pore (#3421; Corning) Transwells. Chemokines (CCL19 [0.45 μg mL^−1^, #250-27B-5UG; PeproTech], CCL21 [1 μg mL^−1^, #250-13-5UG; PeproTech], or CXCL12 [270 ng mL^−1^, #250-20B-2UG; PeproTech]) stabilized with BSA or no chemokine (BSA alone; control) in 600 μL of PBS was added to the bottom of the Transwell. Equal amounts of BSA concentration were provided in the PBS of both top and bottom to prevent migration due to BSA. Transwells were incubated for 2 h at 37°C with 5% CO_2_ before migrated cells were harvested, stained, and analyzed by flow cytometry. SP or M2-SP phenotype and cell counts were quantified using flow cytometry gating and a semi-mature staining panel. The resultant migrated cell number was divided by the corresponding loaded cell number and normalized to *Kmt2d^+/+^* controls under BSA conditions.

### TREC analysis of RTE

Within developing T-cells, rearrangement of the gene segments encoding the T-cell receptor (TCR) occurs, resulting in chromosomal sequences that are excised to produce episomal DNA by-products called TRECs. TRECs are stable, not duplicated during mitosis, and diluted out with each cellular division; therefore, TRECs serve as markers for RTEs in the peripheral blood. To detect RTE, TREC numbers are analyzed from peripheral blood and normalized to the T-cell numbers from the blood *via* flow cytometric gating. Briefly, peripheral whole blood was collected in K_2_EDTA Microtainer tubes (BD; #365974) or with 50 μL – 100 µL of sodium heparin–coated tubes (#540-15; Fresenius Kabi, Lake Zurich, USA), subsequently stained with CD45-PE-Cy7 (#368531; BioLegend) or CD45-Alexa700 (#103113; BioLegend) and CD3-FITC (#130-080401; Miltenyi Biotec), fixed/lysed with Cytofix/Cytoperm (554722; BD), and stored in 1× PBS until run on the flow cytometer (BD LSR Fortessa/BD FACSverse). The gating percentage was used to normalize the data to CD45^+^ CD3^+^ total T-cells. DNA from 100 µL of whole blood was extracted using a QIAamp DNA micro kit per manufacturers’ directions and quantified optically by Qubit 2.0 Fluorometer, as done in the “Recombination Assessment of Cre” section. A PCR reaction was performed using 12.5 – 40 ng of extracted DNA per reaction and PowerUp SYBR Green Master Mix (#A25742; Applied Biosystems). Mouse primers for Ca were used as an internal control (Mouse Ca Forward: 5′-TGACTCCCAAATCAATGTG-3′; Mouse Ca Reverse: 5′-GCAGGTGAAGCTTGTCTG-3′), whereas mouse primers for TREC were used to quantify TREC (Mouse sjTREC Forward: 5′-CCAAGCTGACGGCAGGTTT-3′; Mouse sjTREC Reverse: 5′-AGCATGGCAAGCAGCACC-3′). Cycling was as follows: 2 min at 50°C, 2 min at 95°C, 40 cycles of: 15 s at 95°C and 1 min at 60°C. After the CTs were extracted, the differences in CT (delta CT) from Ca and sjTREC were calculated and run through the equation sjTREC/percent CD45^+^CD3^+^ cells to normalize to the number of T-cells.

### RNA isolation, library preparation, and sequencing

Isolated *Kmt2d-*KO and littermate control thymic cells were centrifuged to pellet, resuspended in 300 µL of Protection Buffer (Monarch RNA Isolation Kit, #T2010S; New England Biolabs Inc. (NEB), Ipswich, USA) and stored at −80°C until isolation. RNA was subsequently isolated following the manufacturer’s instructions of the Monarch RNA Isolation Kit (#T2010S; NEB). RIN scores and concentration were identified using High Sensitivity RNA ScreenTape (#5067-5579/#5190-6507; Agilent Technologies, Santa Clara, USA) and run using the Agilent Technology software with an electronic ladder. Quality RIN scores above 7 were used for RNA-Seq library preparation. Samples were stored at −80°C until library construction. Library construction (input: 40 ng per CD8^+^SP sample) was performed using a NEBNext Poly(A) Magnetic Isolation Module (#E7490; NEB) followed by a NEBNext Ultra II Directional RNA library prep kit for Illumina (#E7760 and/or #E7770 with #E7765; NEB) with size selection by AMPure XP (#Ab3881; Beckman Coulter, Brea, USA) beads, according to manufacturers’ protocols. Library quantification and quality checks were done using KAPA Library Quantification Kit for Illumina (#KK4824; Kapa Biosystems, Cape Town, South Africa), High Sensitivity D1000 DNA Kit on BioAnalyzer (#5067-4626/#5190-6502; Agilent Technologies). Paired-end 50-bp read sequencing was obtained for pooled libraries using a NovaSeq 6000 instrument (Illumina, San Diego, USA).

RNA-seq analysis: Transcriptomic data collected by RNA-Seq were analyzed to determine the genes present in each sample, their expression levels, and the differences in expression levels between *Kmt2d* ^-SET-fl/fl^ *Cre^+^* conditional mutant and *Kmt2d* ^-SET-fl/fl^ control (CD4^+^SP *Lck*-Cre^Mar^, CD8^+^SP *Lck*-Cre^Mar^, CD8^+^SP *Vav1*-iCre). We also utilized publicly accessible data for CD8^+^SP from *Lck*-Cre *Dot1l* ^fl/fl^ GEO accession: GSE138910 and for *CD4*-Cre *Kmt2d* ^-exon16-19-fl/fl^ KO naïve CD4^+^ (GEO: GSE69162) and naïve CD8^+^ peripheral T-cells (NCBI SRA: PRJNA541991). RNA-Seq analysis (from our thymocytes) was performed using three methods. The results of the analysis from the first analysis program, SciDAP (https://scidap.com/), are used for the figures; the two alternative methods (Salmon [2] and HISAT2 [3] alignment tools) were used for verification (data not shown).

Before uploading into SciDAP, two files from each lane were merged into the same file (R1 and R2 merged; L1 and L2 merged). Within the bioinformatics website SciDAP, we imported the merged FASTQ files, trimmed adapters from the files (https://github.com/datirium/workflows/blob/master/workflows/trim-rnaseq-pe.cwl), aligned reads to the mouse genome mm10 (25) and calculated read numbers and RPKMs for transcripts, as previously described (26, 27). Differential expression analysis was performed by DESeq2 (https://github.com/datirium/workflows/blob/master/workflows/deseq.cwl). Each set of data (*Kmt2d* ^-SET-fl/fl^ *Cre^+^)* with their corresponding littermate controls (*Kmt2d* ^-SET-fl/fl^ *Cre^−^*) were run separately using pairwise DESeq2 (i.e., 1. CD4^+^SP *Lck*-Cre^Mar^, 2. CD8^+^SP *Lck*-Cre^Mar^, 3. CD8^+^SP *Vav1*-iCre). Additionally, publicly available datasets were analyzed using pairwise DESeq1 or DESeq2 pipelines (control versus experimental) and subsequently compared with our gene lists. Data files were exported from SciDAP and filtered within Microsoft Excel using the following criteria: 1. For thymocyte KMT2D-regulated genes: RPKM ≥5 in at least one condition, adjusted *P*-value ≤ 0.01, and Log2 fold change ≥0.59 or ≤−0.59 to be considered “upregulated” or “downregulated,” respectively, in wild-type compared with *Kmt2d*-deficient/mutant thymocytes; 2. For peripheral KMT2D-regulated activation genes: a. RPKM ≥5, *P*-value ≤0.99, Log2 fold change ≥0.59 in either *activated* sample of peripheral control and *Kmt2d*-KO cells when compared with unstimulated and b. RPKM ≥5 in either sample, *P*-value ≤0.99, and Log2 fold change ≤−0.59 in *Kmt2d*-KO when comparing between wild type and KO). Gene visualization (upregulated/downregulated vs. control littermates) is displayed *via* Volcano plot (VolcaNoseR; https://goedhart.shinyapps.io/VolcaNoseR/ (28); **Supplementary Figure 3**). The resulting KMT2D-regulated and KMT2D-regulated activation lists were placed into a Venn diagram (Venny v2.1; https://bioinfogp.cnb.csic.es/tools/venny/index.html (29)) to determine the overlapping KMT2D-regulated genes between the datasets. The resulting overlapping genes were copied into ToppGene Suite (https://toppgene.cchmc.org/ (30–33)). Using the program ToppFun with the manufacturer’s recommended settings and analyzed for biological gene ontology “GO” terms (with cutoffs being false-discovery rate Benjamini and Yekutieli [FDR B&Y] = 0.05).

For integrin heatmaps from the datasets: All individual sample RPKM values were run using the Feature expression merge pipeline, a program that combines RPKM expression from several experiments, from SciDAP. The heat maps were generated *via* importing a table of the integrin RPKMs generated from Feature expression merge pipeline selected in Microsoft Excel into Morpheus (https://software.broadinstitute.org/morpheus). Heatmap scales were based on overall RPKM for the heatmap with cutoffs 0 (black) 5/10 (blue), 25 (cyan), 50 (green), 100 (yellow), 250 (red), and 500 (white) for a gradient coloring to reflect the changes.

RNA-Seq analysis alternative (for confirmation): For Salmon (2) and HISAT2 (3) alignment tools, following quality checking with the software FastQC, reads were trimmed with trim-galore/0.5.0 to remove reads with adaptors.

(2) Salmon alignment tool. We built a mouse index using FASTA file of all mouse cDNA sequences downloaded from Ensembl (ftp://ftp.ensembl.org/pub/release-91/fasta/mus_musculus/cdna/Mus_musculus.GRCm38.cdna.all.fa.gz) and pseudo-mapped the RNA-Seq reads with Salmon (v1.1). Subsequently, resulting transcript quantifications were imported into R to get gene-level counts, using the tximport R package. The differential analysis was performed with DESeq2, retaining counts greater than 10.

(3) HISAT2 alignment tool. We built a mouse index using FASTA file downloaded from Ensembl (ftp://ftp.ensembl.org/pub/release-101/gtf/mus_musculus/Mus_musculus.GRCm38.101.gtf.gz) and mapped the reads with the alignment tool HiSat2/2.1.0. The generated Sam files were processed using Samtools/1.9. The reads mapped to feature (exon) and meta-feature (gene) were counted with the feature Counts function from Subread/1.6.3 using an annotation file downloaded from Ensembl (ftp://ftp.ensembl.org/pub/release-101/gtf/mus_musculus/Mus_musculus.GRCm38.101.gtf.gz). This count matrix served as the input for the differential analysis, which we performed using DESeq2. For (2) and (3): Differentially expressed genes were further analyzed for the enriched pathways with an online tool, WEB-based Gene SeT AnaLysis Toolkit (webgestalt.org).

### Visualization of *Kmt2d* ^-exon16-19-fl/fl^ KO peripheral naïve CD8^+^ T-cells genes at a single-cell level

Using SciDAP, we ran a multistep single cell (sc) RNA-Seq pipeline on the GEO accession: GSE217656 (the stimulated and unstimulated samples were run separately, only stimulated dataset results are shown) (12). First, the feature-barcode matrices were generated by Cell Ranger Count using the mouse (mm10) genome as a reference (https://github.com/datirium/workflows/blob/master/workflows/single-cell-preprocess-cellranger.cwl). Next, the multiple datasets were merged with Cell Ranger Aggregate (https://github.com/datirium/workflows/blob/master/workflows/cellranger-aggr.cwl) without normalization. Then, the merged datasets were filtered to remove low-quality cells (excluding those with low [<2k] UMI or high [>3k] gene number; https://github.com/datirium/workflows/blob/master/workflows/sc-rna-filter.cwl). During the dimensionality reduction step, the datasets were normalized (sctglm) and scaled with Seurat and integrated with Harmony using default parameters (https://github.com/datirium/workflows/blob/master/workflows/sc-rna-reduce.cwl). The dataset was clustered (https://github.com/datirium/workflows/blob/master/workflows/sc-wnn-cluster.cwl) to yield the scRNA plot viewable *via* UCSC cell browser (34). Top genes from each cluster were both automatically and manually used to classify each peripheral naïve CD8^+^ T-cell subpopulation. The relative gene expression heatmap for the selected genes was generated with the dittoSeq R package (35). The scRNA heatmap displays gene expression across each noted integrin gene (row), with each line representing a cell’s relative expression. Cells are grouped by their classified cluster (shown above heatmap). Relative scale: low (black) to high (yellow).

### Proposed transcription factor binding at promoter region of KMT2D downregulated genes

To understand potential transcription factors binding within the promoter region of downregulated genes in the following variants: 1. CD4^+^SP *Lck*-Cre^Mar^, 2. CD8^+^SP *Lck*-Cre^Mar^, and 3. CD8^+^SP *Vav1*-iCre compared with their littermate controls (*via* DESeq2 criteria), we used the defined upstream 1 kb from the transcriptional start site (promoter region) locations from our filtered downregulation in variant gene lists and within SciDAP using the Motif Finding with HOMER with random background regions (36). The two resulting transcription factor binding motif lists (known and *de novo*) from each dataset were generated. The proposed transcription factors were filtered against our DESeq2 RPKM values for expression. These transcription factors were compared between known and *de novo* for each dataset for validation and compared across datasets. To determine if a transcription factor binding motif is true (not an artifact), the motif is required to be consistently enriched across the models and contain the length/complexity needed to accurately assign.

### Chromatin immunoprecipitation

The IVG genome browser (37, 38) (accessed *via* SciDAP Trim Galore ChIP Seq Pipeline Single-Read; mm10) was used to locate KMT2D binding in *cis*-regulatory regions (and display MACS called peaks) of the integrin and egress licensing genes (*Itgae*, *Itgal*, *Itgb2*, *Itgb7, Itgb1*, and *Klf2*) around the transcriptional start site (TSS) and into the gene body in naïve CD4^+^ T-cells from a publicly accessed GEO accession: GSE69162. ChIP-Seq file SRR2037221 was analyzed using corresponding input file SRR2037222. An additional H3K4me1 ChIP-seq analysis (on GEO accession: GSE69162) was performed in unstimulated control or *Kmt2d*-KO peripheral naïve CD4^+^ T-cells. The total mapped reads for all ChIP-Seq samples were normalized to 1 million reads.

### Chromatin immunoprecipitation–polymerase chain reaction

Thymi were harvested and single-cell suspensions were created as described above. As previously described by Sailaja et al. (39), 8 × 10^7^ total thymocytes were cross-linked with 1% formaldehyde in PBS with 0.5% bovine serum albumin at room temperature for 10 min, followed by the addition of glycine in water solution to a final concentration of 125 mM glycine for 5 min. After two washes with ice-cold PBS and their subsequent centrifugations at 300 × *g* for 7 min each, the cells were lysed with ChIP-Lysis buffer (10 mM EDTA, 1% [w/v] SDS, 50 mM Tris–HCl, pH 7.5, one tablet of Protease Inhibitor Cocktail [#11836170001; Roche, Mannheim, Germany], and 0.1 mM phenylmethylsulfonyl fluoride [PMSF], followed by sonication in a Bioruptor water bath of five cycles of 5 min, set on HIGH, 30 s ON, and 30 s OFF). 25 µg of DNA diluted in ChIP dilution buffer (0.01% [w/v] SDS, 1.1% [v/v] Triton, 1.2 mM EDTA, 16.7 mM Tris–HCl, pH 8.1, 167 mM NaCl) was precipitated overnight at 4°C with H3K4me3 antibody (#9727; Cell Signaling Technology, Danvers, USA) or normal rabbit IgG (considered “input,” # 2729; Cell signaling technology). PCR of the precipitated product (5 ng) was performed using a reaction with 5 µL SYBR Green (#A25742; Thermo Fisher Scientific) and 0.5 µL of 10 µM primer listed in **Supplementary Table 3**. Primer sequences were designed based off the 8-week Thymus H3K4me3 Histone Modification by ChIP-seq Signal from ENCODE/LICR located under the track name Thymus H3K4me3 in the Mouse July2007 (NCBI37/mm9) assembly on the UCSC genome browser (40, 41). Primers were designed with the intention that the amplicons would span the H3K4me3 binding peak (**Supplementary Figure 4**). The IVG genome browser (accessed *via* SciDAP Trim Galore ChIP Seq Pipeline Single-Read; mm10) was used to locate KMT2D binding in *cis*-regulatory regions of the egress licensing genes within −1 kb of transcriptional start site (TSS) into the gene body up to 5 kb (first few exons) in naïve CD4^+^ T-cells from a publicly accessed ChIP-Seq file SRR2037221 analyzed using corresponding input file SRR2037222 (37, 38). *In silico* PCR (UCSC genome browser; mm10 genome) was used to generate the primer location (**Supplementary Figure 4** indicated in blue) from the ChIP-PCR primer sequence designed with the H3K4me3 binding peak (40).

### Assessment of gene and protein sequence similarities

Murine ITGAE (Gene ID: 16407; Protein GenBank ID: ABD49099.1), ITGAL (16408; AAI45803.1), ITGB2 (16414; AAI45645.1), and ITGB7 (16421; EDL03997.1) protein and gene sequences were obtained from the NCBI database for genetic and protein similarities. For comparison of the *cis-*regulatory region (1,001 base pairs upstream from the transcriptional start site sequence) was determined using the UCSC genome browser with mm39 alignment, the DNA sequence generated and run through LALIGN Pairwise Sequence Alignment (https://www.ebi.ac.uk/Tools/psa/lalign/; a program that finds internal duplications by calculating non-intersecting local alignments of protein or nucleotide sequences) (42).

### Statistical analysis

For murine flow phenotypic, weight-based, and ChIP-PCR data, significance was determined using a parametric, unpaired, Welch’s corrected *t-test* against the littermate. The *P*-values are noted by asterisks: *P*-value > 0.05 (ns), *P*-value < 0.05 (*), *P*-value < 0.01 (**), *P*-value < 0.001 (***), and *P*-value < 0.0001 (****). RNA-Sequencing was analyzed using DESeq2, which utilizes the Benjamini–Hochberg method to generate *P*-adjusted values.

For human data, significance was determined using the binomial test, with the null hypothesis being that individuals with KS1 have the same probability of falling outside the reference range (2.5 to 97.5 percentile) equal to 5%, that is, the same as healthy age-matched individuals. The resulting P-values were subsequently adjusted for multiple testing with the Bonferroni method and noted by asterisks: P-value > 0.05 (ns), P-value < 0.001 (***), P-value < 1e-9 (^), and P-value < 1e-14 (&).

## Results

### *Kmt2d* loss alters SP thymocyte progression in a dosage-dependent manner

To understand the role of KMT2D across T-cell development, we first evaluated the gene’s thymic function using multiple murine models that span from systemic to T-cell-specific and from constitutive to conditional to help define valid consistent changes caused by *Kmt2d* deficiency and rule out model-contributed artifacts. To do this, we employed a constitutive *Kmt2d* haploinsufficiency (herein called *Kmt2d^+/βgeo^* mice) (19), which reportedly profiles similar to human KS1. We validate the findings utilizing two conditional T-cell-specific *Kmt2d* KOs (*CD4*-Cre, *Lck*-Cre^Mar^) systems known to initially recombine at distinct late DN thymocyte stages (**Supplementary Figure 1**) to help narrow down the *Kmt2d* influences intrinsic to T-cells.

**Figure 1:**
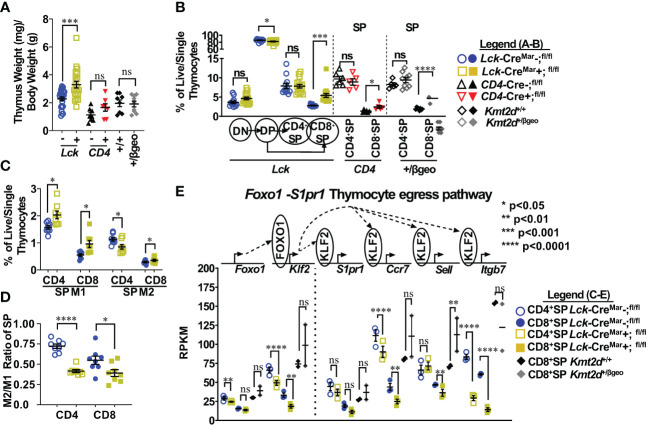
Shifts toward enhanced CD8^+^SP cell percentage, accumulation of Mature 1 SP cells, and alterations in the *Foxo1* licensing egress pathway at the population level in *Kmt2d*-deficient mice. **(A)** Organ weight of thymus (milligrams) normalized to body weight (grams). **(B)** Flow cytometric analysis of thymocyte populations across development, as indicated: double-negative (DN), double-positive (DP), and single-positives (CD4^+^SP, CD8^+^SP) as a percent of total live/single thymocytes. *Lck-*Cre^Mar^; *Kmt2d* ^-SET-fl/fl^ thymocyte populations (left) and CD4^+^SP and CD8^+^SP from *CD4-*Cre; *Kmt2d* ^-SET-fl/fl^ (middle) and *Kmt2d^+/βgeo^* (right) separated by dotted lines. CD8^+^SP are significantly enhanced in all models. (C/D) Thymic *Lck-*Cre^Mar^; *Kmt2d* ^-SET-fl/fl^ CD4^+^SP (open) and CD8^+^SP (closed) maturation by flow cytometry, progressing from Mature 1 MHCI^+^CD69^+^CCR7^+^TCRβ^+^ (M1; left) to MHCI^+^CD69^-^CCR7^+^TCRβ^+^ Mature 2 (M2; right) as a percent of total thymocytes **(C)** or by M2-SP/M1-SP ratio using values from % of total thymocytes values **(D)**. Each marker displays average cells from individual mice (n = 2–15; from two or more independent experiments/condition). **(E)** Disruption of the *Foxo1* egress licensing pathway expression (RPKM) in *Kmt2d*-deficient CD4^+^SP and CD8^+^SP thymocytes. Pathway depicted above graph: FOXO1 protein binds to promote gene expression of *Klf2*, fueling KLF2 protein expression (protein depicted within circle), which binds to four downstream genes inducing increased RNA expression of the stated genes. Relevant genes are displayed on the top of each dataset. Each marker displays average cells from individual mice (n = 2–15) and black lines represent mean ± SEM. The mouse lines are displayed on the graphs: *Kmt2d^+/βgeo^* (^+/+^ black diamonds/*^+/βgeo^* gray diamonds), *Lck*-Cre^Mar^; *Kmt2d*-^SET-fl/fl^ (Cre^−^ blue circles/Cre^+^ yellow squares), and *CD4*-Cre; *Kmt2d*-^SET-fl/fl^ (Cre^−^ black up-right triangles/Cre^+^ red inverted triangles). For all cellular/organ phenotype analysis, we employed parametric, unpaired, Welch’s corrected *t-test* (mutant compared with corresponding littermate) for significance. For **(E)**, DESeq2 generated adjusted *P*-values using the Benjamini–Hochberg method. Significance is listed on graph based on *P*-value or DESeq2 adjusted *P*-values (e) > 0.05 (ns), *P*-value < 0.05 (*), *P*-value < 0.01 (**), *P*-value < 0.001 (***), and *P*-value < 0.0001 (****). Multiple independent experiments were used for **(A-D)**.

First, we analyzed murine thymus weight and found no significant change in *Kmt2d^+/βgeo^* mice, nor in *CD4*-Cre-driven *Kmt2d* KOs; however, the T-cell-specific *Lck*-driven *Kmt2d*-KO led to a significant increase in thymus weight (**Figure 1A**;**Supplementary Figure 5A**). Next, to elucidate the stages in which *Kmt2d* plays a role during thymocyte development, we analyzed thymocyte populations (CD4^-^CD8^-^DN, CD4^+^CD8^+^DP, CD8^+^SP, and CD4^+^SP) across developmental time. Strikingly, *Kmt2d* loss significantly increased the percentage of CD8^+^SP, but not CD4^+^SP thymocytes (of live/single cells) in both *Kmt2d*-KO models and in the *Kmt2d*-haploinsufficent model (**Figure 1B**).

Thymocyte SP maturation occurs concurrently with negative selection, proceeding from semi-mature SP cells into the “non-licensed” Mature 1 (M1)-SP stage and then onto the Mature 2 (M2)-SP thymocyte stage, where cells are licensed, selected, and become ready for egress. We selected *Kmt2d*-KO thymi to further characterize *Kmt2d*’s influence on SP maturation as deletion of both alleles may allow us to more clearly view the mechanism without potential compensation. By flow cytometry, *Kmt2d*-KO (*Lck*-Cre^Mar^) thymocytes have a significantly increased percentage of M1-SP (**Figure 1C**). In addition, CD44 cleavage (which is required for SP maturation and egress) (43) did not occur as frequently within either *Kmt2d*-KO or -haploinsufficient SP cells as their control littermates (**Supplementary Figure 5B**). When evaluating the SP population, we found that the T-cell-specific *Kmt2d* KO mice most likely had a partial block in the transition between M1 to M2 differentiation as we observed a relative M1 accumulation and a M2 reduction, but the overall CD4^+^SP population does not change as a percentage of total thymocytes (**Figures 1B–D**). In contrast, CD8^+^SP demonstrated an increase in both M1-SP and M2-SP thymocyte populations leading to an increase in the overall percentage of the CD8^+^SP population in the overall thymocyte pool (**Figures 1B–D**). In contrast, *Kmt2d* haploinsufficient mice do not have the M1-SP accumulation but rather a trend toward increased M2-SP (**Supplementary Figures 5C, D**). These heterogenous SP changes demonstrate *Kmt2d*’s differential effect on CD4^+^SP and CD8^+^SP thymocytes as they transition from M1 to M2 or in the ability of M2 cells to egress.

SP differentiation has previously been partially characterized, but the role of *Kmt2d* in this process is unknown. Reportedly, the transcription factor, FOXO1, is only expressed in the most mature SP cells (CD69^−^ and no longer receiving negative selection TCR signaling) and normally binds at the *Klf2* locus *(* 44–46). Subsequently KLF2 regulates the transcription (through binding) of a plethora of homing genes including *S1pr1* (encodes sphingosine-1-phosphate receptor 1, the receptor for S1P1), *Itgb7*, *Ccr7*, and *Sell* (encodes CD62L), all of which are normally upregulated during the egress process (44–47). As M1-SP thymocyte accumulation was observed in *Kmt2d*-KO SP thymocytes, we investigated whether dysregulation of the FOXO1-driven egress licensing pathway could be a potential culprit. In *Kmt2d*-KO SP thymocytes, we observe either a significant or trending reduction in expression of nearly all FOXO1 target genes analyzed (**Figure 1E**). Both phenotypic and RNA analyses revealed a population level maturation deviation in *Kmt2d-*KO SP. In contrast, *Kmt2d*-haploinsufficient CD8^+^SP thymocytes did not have the reduced expression but rather a trend of enhancement of *Foxo1*, *Klf2*, *S1pr1*, and *Ccr7* (**Figure 1E**). Interestingly, *Kmt2d*-haploinsufficient CD8^+^SP thymocytes displayed an independent reduction of *Itgb7* expression, albeit not significant (**Figure 1E**). The FOXO1 pathway may require at least one allele of *Kmt2d* for function, as complete *Kmt2d* loss resulted in reduced late maturation (M2), whereas one allele is sufficient to support normal expression of this pathway. As the CD8^+^SP population was relatively expanded and *Itgb7* reduction within SP thymocytes is conserved across all *Kmt2d*-deficient models, two alleles of *Kmt2d* may be required for *Itgb7* expression (potentially by direct regulation) and *Itgb7* loss may contribute to CD8^+^SP accumulation (**Figure 1E**).

To further understand KMT2D’s broader regulatory function in thymocyte development, we evaluated gene expression changes in our models and characterized overall biological impacts. Specifically, we evaluated significantly downregulated genes in the bulk *Kmt2d* KO SP thymocytes that were shared between the CD4^+^SP and CD8^+^SP in different conditional KO models (*Lck*- vs. *Vav1*-driven Cres; **Supplementary Figure 3**). KMT2D is a transcriptional co-activator that regulates gene activation through histone H3K4 methylation on promoters and enhancers; therefore, *a priori* one would expect direct KMT2D-targets to show reduced RNA expression in *Kmt2d*-KO compared with control cells. Downregulated genes (**Supplementary Table 4**) that overlapped in the different SP KO models clustered in the biological gene ontology terms “cell adhesion,” “GTPases,” and “activation,” which are all downstream functions of integrins. Similarly, the biological function of genes downregulated in *Kmt2d* haploinsufficient thymocytes (DN1 and CD8^+^SP) and CD8^+^SP KO thymocytes overlapped with “cytotoxic (effector),” “response/activation,” and “migration” gene ontology terms, but CD8^+^SP KO cells additionally had downregulated genes associated with “proliferation” and “differentiation.” Together, these findings suggest that *Kmt2d* loss in distinct models affects similar pathways of activation and adhesion/migration.

### KMT2D regulates integrin expression during thymocyte development

Since the biological categories associated with KMT2D aligned with activation and adhesion, we next investigated integrin expression in thymocytes. Altered integrin expression, changes in motility and integrin-directed localization, and disrupted cellular signaling have been previously described in various KMT2D deficient model systems (2, 12, 48, 49).

**Figure 2:**
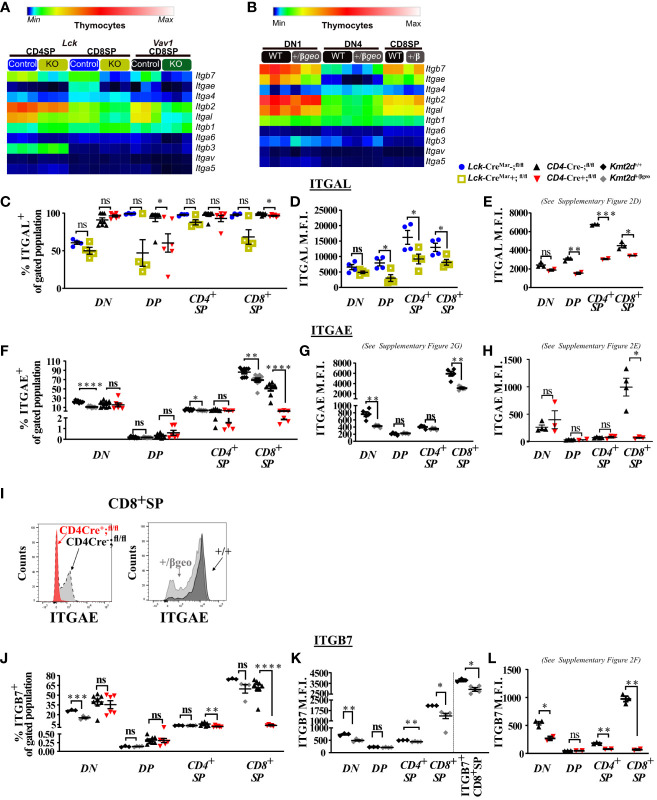
Dysregulated thymic integrin expression in *Kmt2d*-deficient models. **(A, B)** Heatmap panel of relatively expressed integrins from: **(A)** *Lck*-Cre^Mar^ or *Vav1*-iCre driven *Kmt2d* KO and corresponding control SP thymocytes (n = 3 mice [biological replicates]/group) from 1 (*Lck*-Cre) or 2 (*Vav1*-iCre) independent experiments. **(B)** *Kmt2d* haploinsufficient and sufficient thymocytes across stages of thymocyte development (DN1, DN4, and CD8^+^SP) from 1 (CD8^+^SP; n = 2 mice [biological replicates]/condition) or 2 (DN1, DN4; n = 3 mice [biological replicates]/condition) independent experiments. DN1 and DN4 contain results from multiple pooled mice in 1 sample (n = 1) within an independent experiment. Relative expression level scale based on RPKM values of the whole plot (black, 0; blue, 10; cyan, 25; green, 50; yellow, 100; red, 250; white, 500). **(C-L)** Flow cytometric population shifts across the thymocyte development: shown as percent positive of indicated thymic population: double-negative (DN), double-positive (DP), and single-positive (CD4^+^SP, CD8^+^SP) of *ITGAL* **(C)**, *ITGAE* **(F)**, and *ITGB7* **(J)** or relative mean fluorescence intensity (MFI; in arbitrary units of intensity (a.u.i.); i.e., protein expression level) of *ITGAL* **(D, E)**, *ITGAE* **(G, H)**, and *ITGB7* **(K, L)**. The following mouse lines (*Kmt2d^+/βgeo^* [^+/+^ black diamonds/*^+/βgeo^* gray diamonds, two independent experiments], *Lck*-Cre^Mar^; *Kmt2d* ^-SET-fl/fl^ [Cre^−^ blue circles/Cre^+^ yellow squares, 1 independent experiment] and *CD4*-Cre; *Kmt2d* ^-SET-fl/fl^ [Cre^−^ black up-right triangles/Cre^+^ red inverted triangles, three independent experiments]) are displayed as one marker per individual mouse (n = 2–9 mice [biological replicates]/condition) on the graphs. The percentage graphs **(C, F, J)** display all independent experiments together; however, MFI graphs **(D, E, G, H, K, L)** only display one independent experiment as cytometer settings and instruments were altered. The MFI for the additional independent experiments (with similar MFI directionality) are displayed in **Supplementary Figures 2D–G**. **(I)** Representative flow histogram overlay of *Kmt2d* deficient with corresponding control littermate each from a single mouse (y axis: Counts, as normalized to mode). Depiction of both the shift toward the negative population and decreased expression level of ITGAE in CD8^+^SP (right; *^+/+^* black/*^+/βgeo^* gray, left; *CD4*-Cre; *Kmt2d* ^-SET-fl/fl^ with Cre^−^ black/Cre^+^ red). Each dataset displays black lines, which represent mean ± SEM. Significance labeled on graphs were determined using a parametric, unpaired, Welch’s corrected *t-test* between a mutant and corresponding littermate controls. Significance based on *P*-value > 0.05 (ns), *P*-value < 0.05 (*), *P*-value < 0.01 (**), *P*-value < 0.001 (***), and *P*-value < 0.0001 (****).

Starting in SP thymocytes, we evaluated integrin gene expression in *Kmt2d* sufficient and conditionally deficient cells (**Figure 2A**). In this case, we have also employed a conditional hematopoietic-activated *Kmt2d* KO driven by *Vav1*-iCre (22), to assist in confirming consistency of changes. We found that both control and *Kmt2d* KO (*Lck*-Cre^Mar^ and *Vav1*-iCre) SP cells expressed integrin genes previously reported in control SP thymocytes (50), including *Itga4, Itga5, Itga6, Itgae* (only CD8^+^SP)*, Itgal, Itgav, Itgb1, Itgb2*, *Itgb3*, and *Itgb7*; however, expression levels did vary by genotype. In *Kmt2d* KO SP thymocytes, genes that produce integrin subunits whose proteins heterodimerize together, such as the *Itgae/Itgb7* and *Itgal*/*Itgb2* pairs, had significantly reduced expression of both alpha and beta genes (**Figure 2A**). Furthermore, *Kmt2d* KO SP thymocytes had reduced expression of two other integrins, *Itga4 and Itga6*, while showing increased *Itgb1* and *Itgb3* expression (**Figure 2A**) compared with controls.

We next analyzed integrin levels in *Kmt2d* haploinsufficient thymocytes across key developmental states (DN1, DN4, and CD8^+^SP populations). Although the DN1 and CD8^+^SP populations of *Kmt2d^+/βgeo^* showed a trend toward reduced leukocyte-specific integrin expression levels, the observed differences were not statistically significant (**Figure 2B**). Furthermore, DN4 thymocytes had modest integrin expression with no significant differences between genotypes (**Figure 2B**). Overall, KMT2D appears to be important for leukocyte-specific integrin expression in SP cells in a gene dose-dependent fashion.

To determine if integrin gene expression changes altered their protein expression levels, we evaluated a panel of differentially expressed integrins (ITGAL, ITGAE, and ITGB7) by flow cytometry across thymocyte developmental time. Our results indicate that the majority of all thymocytes express ITGAL with its expression increasing across developmental time (lowest expression in DN, enhanced in DP, with highest expression in SP; **Figures 2C–E**). When comparing *Kmt2d* KO mice with control mice, a significant decrease in ITGAL expression levels (decreased geometric mean fluorescence intensity; MFI) begins in the DP stages and continues in both SP thymocyte populations (**Figures 2D, E**; **Supplementary Figure 2D**). Additionally, changes in ITGAL positivity (shifts in population positive to negative ITGAL expression) in *Kmt2d*-targeted mice trend toward decreased ITGAL populations in both DP and CD8^+^SP thymocytes compared with control mice (**Figure 2C**). In contrast, both the ITGAE^+^ population and ITGAE MFI are specifically decreased in the CD8^+^SP thymocytes, but not in DP cells in *Kmt2d*-deficient models (**Figures 2F–I**; **Supplementary Figures 2E, G**). Similarly, ITGB7 displayed similar patterns of reduced MFI and populations shifts in CD8^+^SP, without altered changes in the DP population (**Figures 2J–L**). In CD8^+^SP, the expression intensity of ITGAE and ITGB7 in control cells is at least twofold higher than other thymocyte populations (DN, DP, CD4^+^SP), whereas in the *Kmt2d* KO CD8^+^SP thymocytes, the ITGAE and ITGB7 MFIs decrease to levels similar to those of DP and CD4^+^SP thymocytes (**Figures 2H, L**; **Supplementary Figures 2E, F**). Lastly, the ITGB7 MFI levels in CD4^+^SP and CD8^+^SP thymocytes within haploinsufficient models were decreased (**Figure 2K**). A closer analysis of CD8^+^SP ITGB7-positive thymocytes reveal that the intensity decrease in the *Kmt2d*-haploinsufficient model specifically corresponds to changes at both the expression level within the positive population and in overall ITGB7 positivity, similar to alterations observed in ITGAE (**Figures 2I–K**). Thus, KMT2D appears to influence this specific group of integrin signaling molecules, and these observed expression abnormalities translate into a measurable effect at the protein level. From the expression and protein integrin subunit shifts, our data support a model where within control SP thymocytes, KMT2D is associated with the expression of integrin dimers ITGAE-ITGB7 and LFA1 (ITGAL-ITGB2), whereas in models deficient in *Kmt2d*, these heterodimers are no longer expressed or are greatly reduced.

### Conserved integrin expression consistent with direct KMT2D control

**Figure 3:**
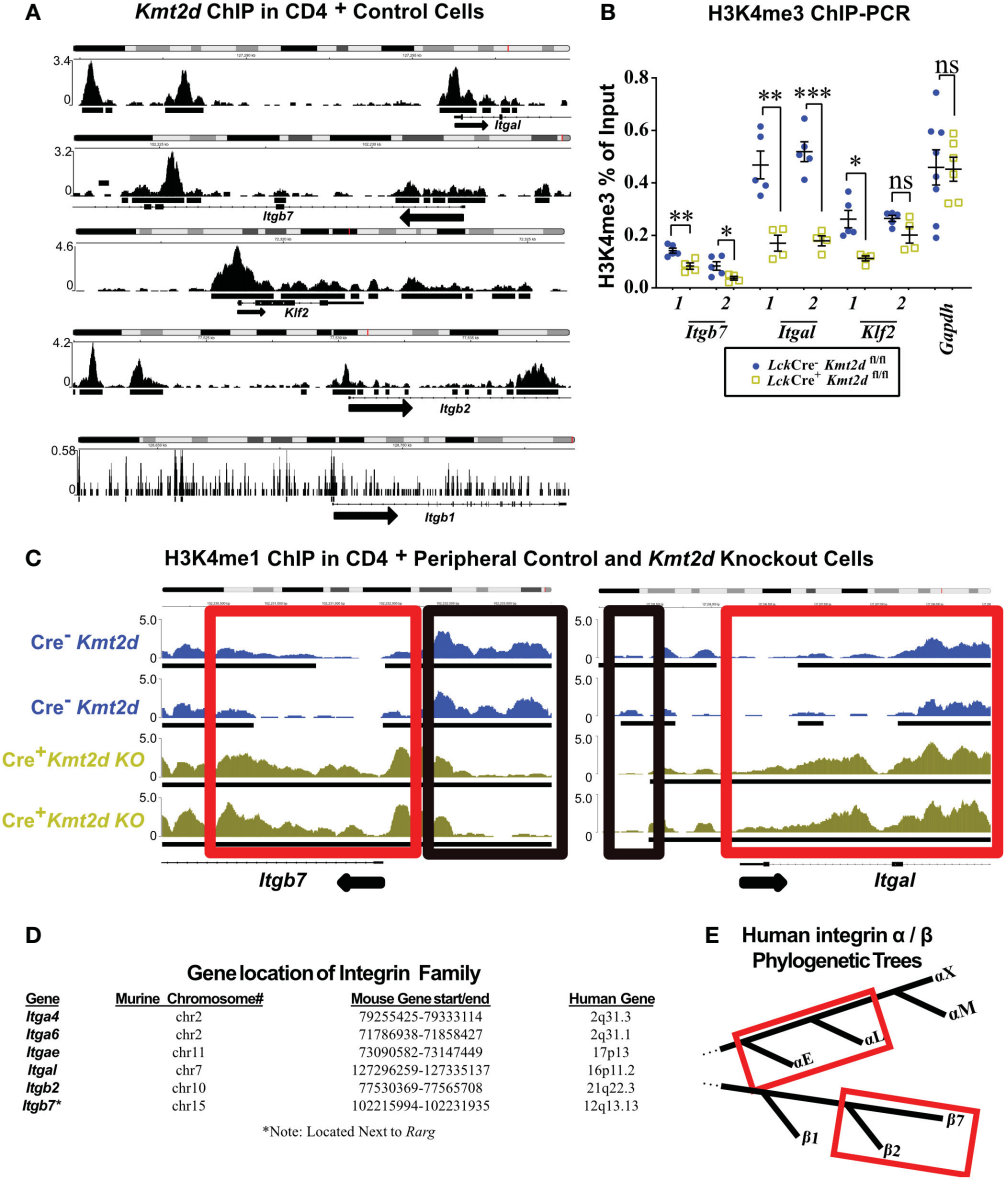
Differentially expressed genes have KMT2D binding sites in *cis*-regulatory regions and demonstrate an abnormal chromatin signature. **(A, C)** Integrative Genomics Viewer browser images of chromatin immunoprecipitation (ChIP) binding of KMT2D **(A)** or H3K4me1 **(C)** in control naïve peripheral CD4^+^ T-cells at *cis*-regulatory regions around the transcription start site (TSS) region of the *Kmt2d* KO downregulated genes from GEO accession number: GSE69162. **(C)** ChIP of H3K4me1 at *cis*-regulatory regions (red and black boxes highlighting regions with shifts between control [Cre^−^, blue] and *Lck*-Cre^Mar^; *Kmt2d* ^-SET-fl/fl^ [Cre^+^, yellow] landscapes) of *Itgb7* and *Itgal* in naïve peripheral CD4^+^ T-cells. Black arrow notes TSS start site and direction for the gene. **(B)** ChIP-PCR of H3K4me3 *Lck*-Cre^Mar^; *Kmt2d* ^-SET-fl/fl^ (Cre^-^ blue circles/Cre^+^ yellow squares; n = 4–8; from three to four independent experiments) bulk thymocyte binding to indicated *cis*-regulatory regions (primer locations displayed in **Supplementary Figure 4**) of *Klf2*, *Itgb7*, and *Itgal* genes, as compared with that of housekeeping genes (*Gapdh*). Each dataset displays black lines, which represent mean ± SEM. Significance determined using a parametric, unpaired, Welch’s corrected *t-test* between a mutant and corresponding littermate controls is listed on graph based on *P*-value > 0.05 (ns), *P*-value < 0.05 (*), 0.01 (**), and 0.001 (***). **(D)** Downregulated integrins and their murine and human chromosomal locations. No observed clustered co-localization of downregulated leukocyte-specific receptor integrin genes on both mouse and human chromosomes, but similar location of *Itga4* and *Itga6*. **(E)** Red box highlighting the proximity of the leukocyte-specific receptor integrins on their localized regions of their respective separate phylogenetic tree regions.

To examine whether KMT2D directly regulates SP thymocyte integrin and egress genes, we utilized published KMT2D chromatin immunoprecipitation-sequencing (ChIP-Seq) data (GEO accession: GSE69162) to identify KMT2D peaks present in putative *cis*-regulatory regions of an SP licensing transcription factor (*Klf2*) and integrins (*Itgal, Itgb2*, *Itgb7*, and *Itgb1*) within peripheral CD4^+^ T-cells (12, 13). In the ChIP-Seq datasets, we observed KMT2D binding in unstimulated peripheral CD4^+^ T-cells at *Itgal*, *Itgb2*, *Itgb7*, and *Klf2 cis*-regulatory regions, but lower reads in *Itgb1* (**Figure 3A**). Next, we selected *Klf2*, *Itgal, Itgb7*, and a control housekeeping gene, *Gapdh*, *cis*-regulatory regions (**Supplementary Figure 4**) for H3K4me3 ChIP-PCR to assess KMT2D catalytic activity, and thus reveal potential direct regulation of KMT2D. We found significantly decreased H3K4me3 levels at *Klf2*, *Itgal*, and *Itgb7 cis*-regulatory regions, whereas the *Gapdh* promoter showed no change (**Figure 3B**). This finding suggests direct KMT2D-catalyzed chromatin modification at these loci. Next, we compared H3K4me1 levels between unstimulated control and *Kmt2d* KO CD4^+^ T-cells in the ChIP-Seq dataset (GEO accession: GSE69162) (12, 13). Normally, H3K4me3 is present at active promoters; however, without KMT2D catalytic function, histone H3K4me2 and H3K4me3 methylation may be less efficient, thus retaining H3K4me1 at promoters of genes that KMT2D can directly regulates. In fact, we observe a significantly enhanced fold change of H3K4me1 reads at the *Itgb7* promoter using MAnorm analysis (**Figure 3C**). Together, these data suggest that KMT2D directly regulates *Itgb7*.

As multiple integrins demonstrated similar alterations in expression upon loss of *Kmt2d*, we hypothesized that they might be co-regulated. KMT2D thymocyte integrin co-regulation did not appear to involve genomic domain regulation as chromosomal location/loci (both murine and human) were not shared by affected integrin genes (**Figure 3D**). Phylogenetically, human α- and β-leukocyte-specific integrins were most similar within their respective chains displaying more than 60% protein homology (**Figure 3E**). To assess whether murine leukocyte-specific integrins genes are mechanistically regulated the same, we compared the murine 1 kb promoter sequences and found 29.5% – 49.3% similarity for non-identical alignments for *Itgb2/7* or *Itgae/l* (**Supplementary Table 5**). Additionally, cross-chain analysis revealed similarity in the promoter regions: 46% – 56.9% for *Itgb7/Itgae, Itgb2/Itgae*, or *Itgb2/Itgal*, but interestingly no overlap between *Itgb7/Itgal* (**Supplementary Table 5**). However, expanding our analysis to the 1 kb promoter regions upstream of the consensus transcriptional start site of all putative murine thymocyte *Kmt2d*-regulated genes, we were unable to identify any motifs of a putative KMT2D transcriptional regulator partner from our datasets. These collective findings suggest that KMT2D may alter integrin expression through regulation at shared promoter regions, although the exact transcriptional co-regulator(s) remain elusive.

### KMT2D regulates SP thymocyte migration through LFA1 expression

**Figure 4:**
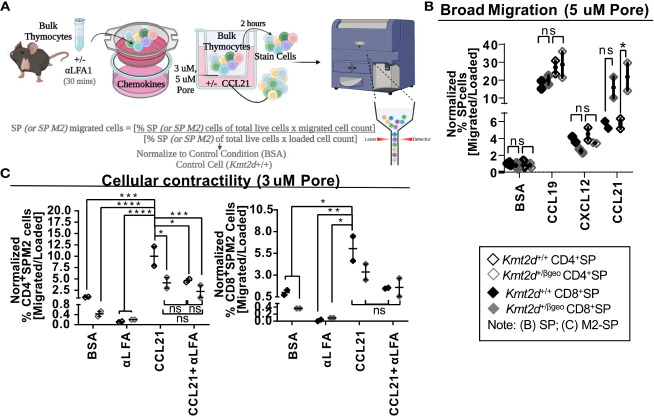
Disruption of contractile motility in *Kmt2d* haploinsufficient SP cells. **(A)** A visualization of the chemotaxis assay procedure: bulk thymocytes are blocked with αLFA1 [or not] and loaded into the top of a 24-well Transwell dish. Created with BioRender.com. Migration is allowed for 2 h at 37°C. Both bulk loaded thymocytes and migrated cells (bottom of Transwell) are harvested and stained for flow cytometric analysis (**Supplementary Figure 2**). Calculated migration of SP. **(B)** or M2-SP **(C)** populations. Resultant migrated cell number was normalized first by the corresponding loaded cell number and then to *Kmt2d^+/+^* controls of BSA condition. **(B)** General motility chemotaxis using a 5μMpore Transwell toward known SP chemokines (CCL19, CXCL12, and CCL21) or **(C)** cellular contractility driven chemotaxis (3μMpore Transwell) of M2-SP toward CCL21 between *Kmt2d* sufficient and haploinsufficient thymocytes (*^+/+^* black/*^+/βgeo^* gray; technical well [**B**] and experimental [**C**] replicates, n = 2–4; representing one independent experiment). The presence of αLFA1 pre-blocking is noted. Each dataset displays black lines, which represent mean ± SEM. Significance labeled on graphs were determined using a parametric, unpaired, Welch’s corrected *t-test* between a mutant and corresponding littermate controls with significance based on *P*-value: *P*-value > 0.05 (ns), *P*-value < 0.05 (*), *P*-value < 0.01 (**), *P*-value < 0.001 (***), and *P*-value < 0.0001 (****).

Chemotactic migration, such as thymocyte migration toward soluble CXCL12 (*via* binding to thymocyte CXCR4) and/or CCL19/21 (both bind to thymocyte CCR7), has been shown to influence migration/egress of SP (23, 24, 51, 52). To functionally test *Kmt2d*’s influence on SP thymocyte migratory capacity, the chemokines CCL19, CCL21, and CXCL12 were added to the bottom of a 5 μM pore Transwell and *Kmt2d* haploinsufficient and control thymocytes were evaluated in regard to un-impeded chemotactic migration (**Figure 4A**). Surprisingly, there was no deficit in un-impeded migration toward CCL19 and CXCL12 chemokines; moreover, there was an enhancement of CD8^+^SP CCL21-driven migration upon *Kmt2d* haploinsufficiency (**Figure 4B**).

SP thymocytes may employ cellular contractility for intrathymic localization, requiring integrin-mediated migration and sensing, interaction with extracellular matrices, and focal adhesion formation. As KMT2D directly regulates integrins and modulates SP differentiation, we evaluated KMT2D’s role in cellular contractility. To do so, we functionally tested cellular contractility migration (3 μM-pore Transwell) (23, 24) in the *Kmt2d* haploinsufficient model. Decreased motility of CD4^+^SP M2-SP *Kmt2d* haploinsufficient thymocytes was observed (**Figure 4C**). Next, we tested if *Kmt2d*-mediated contractile migration requires LFA function. We found CD4^+^SP M2-SP *Kmt2d* haploinsufficient thymocytes had comparable migration deficits to that of LFA1 blocked *Kmt2d* sufficient cells. Furthermore, *Kmt2d* haploinsufficient thymocytes were not significantly altered by the addition of LFA1 blocking antibody. Together, these data suggest the contractile migratory capacity of CD4^+^SP M2-SP thymocytes is dependent on its KMT2D-induced LFA expression.

To further explore possible mechanisms driving the observed cellular contraction defect, we performed gene expression analysis with a focus on the focal adhesion *Mst1* pathway in *Kmt2d* KO cells. *Mst1* drives localization of proteins (such as LFA1) to the leading edge during cellular migration (53), functionally impacting thymocyte migration and potentially influencing overall thymic egress. We evaluated *Mst1’s* downstream pathway (*Rap1a, Dennd1c, Vasp, Itgal, Itgb2*) genes that are required for functional contractility and found many *Mst1*-mediated genes are significantly downregulated in *Kmt2d* KO cells (**Supplementary Figure 5**). Together, these data indicate the potential role of the integrin deficiency, specifically LFA1, in the decreased migration capacity of *Kmt2d* defective cells.

### Peripheral *Kmt2d*-deficient T-cells demonstrate thymic deficiencies and dosage-related peripheral T-cell shifts

**Figure 5:**
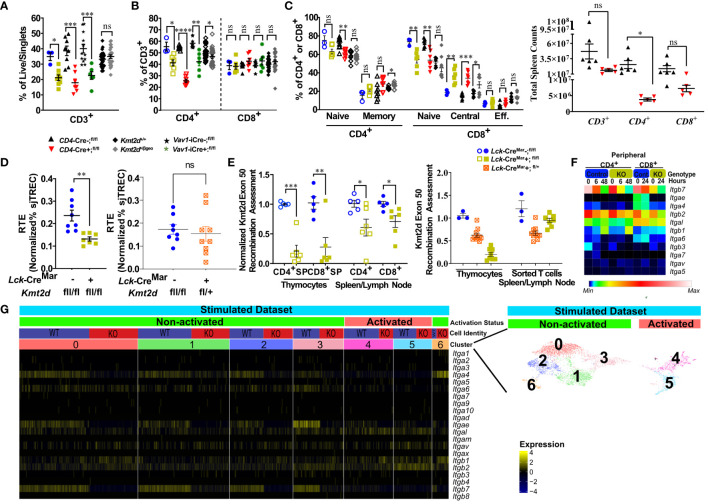
*Kmt2d*-deficient peripheral T-cells demonstrate decreased total T-cells (KOs), shifted naïve-to-memory phenotypes, and consistent deficiencies in leukocyte-specific integrins. **(A)** Flow cytometry of peripheral CD3^+^ as percent of live/singlet cells. **(B)** Percentage of peripheral CD4^+^ (open, left) and CD8^+^ (closed, middle) T-cell populations of overall T-cell (CD3^+^) populations. **(C)** Percentage of peripheral splenic naïve and memory T cells (based on CD62L and CD44 populations) of CD4^+^ (open) and CD8^+^ (closed). **(D)** Total peripheral blood recent thymic emigrant changes shown through sjTREC PCR values normalized to CD3^+^ percent in *Lck*-Cre^Mar^; *Kmt2d* ^-SET-fl/fl^ comparison between control and *Kmt2d* KOs (left) or control and T-cell haploinsufficiency (right) **(E)** Genomic DNA recombination assessment of exon 50 (SET domain), which is cleaved during Cre-recombination at *Loxp3* sites, in thymocytes and peripheral spleen/lymph node CD4^+^ (open) and CD8^+^ (closed) T-cells of *Lck*-Cre^Mar+^; *Kmt2d* ^-SET-fl/fl^ and littermate Cre- controls (left). Calculation plotted is 2^-(Mutant [CT of^ *^Kmt2d^* ^-Exon50/CT of^ *^Kmt2d^* ^-Total R2] – Control [CT of^ *^Kmt2d-^* ^Exon50/CT of^ *^Kmt2d^* ^-Total R2])^, where the KOs are normalized to all *Kmt2d* and then normalized to the average of the cell specific *Lck*-Cre^Mar-^; *Kmt2d* ^-SET-fl/fl^ control; see schematic in **Supplementary Figure 1**. Comparison of *Kmt2d* dosage on recombination in bulk thymocytes and peripheral sorted T-cells (right). **(A-E)** The following mouse lines are displayed as splenic cells (or as listed: thymocytes or peripheral blood [E]) from individual mice (n = 3–30; from two or more independent experiments) on the graphs: *Kmt2d^+/βgeo^* (^+/+^ black diamonds/*^+/βgeo^* gray diamonds), *Lck*-Cre^Mar^; *Kmt2d* ^-SET-fl/fl^ (Cre^-^ blue circles/Cre^+^ yellow squares), *CD4*-Cre; *Kmt2d* ^-SET-fl/fl^ (Cre^-^ black up-right triangles/Cre^+^ red inverted triangles), and peripheral blood *Vav1*-iCre; *Kmt2d* ^-SET-fl/fl^ (Cre^-^ black stars/Cre^+^ green asterisks). Each dataset displays black lines, which represent mean ± SEM. Significance labeled on graphs were determined using a parametric, unpaired, Welch’s corrected *t-test* between mutants and corresponding littermate controls with *P*-values noted by asterisks: *P*-value > 0.05 (ns), *P*-value < 0.05 (*), *P*-value < 0.01 (**), *P*-value < 0.001 (***), and *P*-value < 0.0001 (****). Heatmaps of relative integrin expression from bulk (**F**; relative expression by plot) and single-cell RNA (**G**; relative expression by row [gene]) analyses of stimulated or unstimulated (0 h, 6 h, 48 h [CD4^+^] and 0 h, 24 h [CD8^+^]) *Lck*-Cre *Kmt2d* ^-exon16-19-fl/fl^ KO and control T-cells. **(G)** Relative expression level scale based on RPKM values of the whole plot (black, 0; blue, 10; cyan, 25; green, 50; yellow, 100; red, 250; white, 500). **(F)** Integrated UMAP of naïve peripheral CD8^+^ T-cells from control and *Kmt2d* KO displaying seven subpopulations clustered (differentiated by colors) and further defined as “activated” and “non-activated”. These subpopulations and classified activation status are the numbers listed above the heatmap of integrin alpha and beta genes, which denote relative expression (black, min; yellow max). Expression data represent publicly available data from one independent experiment/condition.

We evaluated the impact of constitutive *Kmt2d* haploinsufficient and conditional *Kmt2d* KO mouse models on the peripheral T-cell compartment. *Kmt2d^+/βgeo^* mice had no significant changes in splenic CD3^+^ T-cell percentages from littermate controls, whereas all three conditional *Kmt2d*-KO models (*CD4*-Cre, *Lck*-Cre^Mar^, and *Vav1*-iCre) showed significant decreases in CD3^+^ T-cells (**Figures 5A, C** **;** **Supplementary Figure 6A**). Across all *Kmt2d-*deficient models, a significant reduction in the CD4^+^ subpopulation percentage was noted, whereas the CD8^+^ subpopulation percentage remained similar to control levels resulting in skewed CD4^+^/CD8^+^ ratios toward CD8^+^ (**Figure 5B** **,** **Supplementary Figure 6B**). Within *Kmt2d*-KO animals, overall CD3^+^CD4^+^ and CD3^+^CD8^+^ populations were decreased (**Supplementary Figure 6A**, **Figure 5C**). Furthermore, we observed reduced naïve and enhanced memory CD8^+^ populations in *Kmt2d*-targeted mouse models (**Figure 5C**).

As our data indicate potential issues with thymic egress, we evaluated RTE abundance at a population level by quantifying sjTREC (54) levels in our mouse models. Within the thymus, the T-cell receptor (TCR) recombination generates excised V(D)J loops (sjTREC), whose concentration is diluted upon each subsequent round of cell division and thus can be quantified at an overall level through peripheral blood analysis. A decreased sjTREC signal could indicate that the peripheral cells either (1) have undergone more cellular divisions than matched control cells; (2) contain a smaller fraction of RTE than control cells; and/or (3) both. We observed a significant decrease in normalized sjTREC signal in *Kmt2d*-KO cells compared with controls, but not in the *Kmt2d* ^-SET-fl/+^ (*Kmt2d*-heterozygous) cells (**Figure 5D**); thus, the models may have differing mechanisms behind their peripheral phenotype.

Next, we evaluated *Kmt2d* locus targeting (i.e., KO) efficiency *via* quantification of Cre-recombinase cleavage of exons 50/51 (SET-domain) in the *Kmt2d* ^-SET-fl/fl^ mice, in both thymocytes and peripheral T-cells (**Supplementary Figure 1**). *Kmt2d*-KO peripheral T-cells showed decreased levels of SET deletion compared with their SP thymocyte precursors (**Figure 5E**). The overrepresentation of peripheral *Kmt2d*-sufficient cells, which appear to have “escaped” thymic CRE recombination, suggests that the complete loss of *Kmt2d* is highly detrimental for thymic egress, proliferation, and/or viability. Of note, in *Kmt2d* ^-SET-fl/+^(*Kmt2d*-heterozygous) thymocytes and peripheral cells have a similar *Kmt2d* recombination efficiency (**Figure 5E**), which demonstrates that one allele of *Kmt2d* is sufficient to permit T-cell egress. The loss of *Kmt2d* KO cells in periphery may be driven by the stark reduction of integrin expression, thymic hypertrophy, and altered thymic SP populations. This contrasts with the *Kmt2d* haploinsufficent model, which had no licensure pathway transcription factor defects, but did have reduced levels of integrins, thus likely limiting but not completely blocking exit. Together, the egress, proliferation, or viability differences between the KO and haploinsufficient models contribute to the observed changes in overall peripheral T-cell numbers and composition.

Integrin expression in peripheral T-cells is required for localization into or retention within tissues. To understand peripheral T-cell integrin transcriptomic expression, naïve peripheral T-cells from available data that employ an alternative *Kmt2d* KO model, which removes exons 16–19 instead of exons 50–51 were assessed at a bulk level (GEO accession: GSE204946 [CD4^+^] and NCBI SRA: PRJNA541991 [CD8^+^]) and then more in depth at a sc level (GEO accession: GSE217656) for relative expression changes before and after stimulation (11, 12). When examined, the presented T-cell figures within the source articles for the *Kmt2d* ^-exon16-19fl/fl^ KO aligned with our own *Kmt2d* ^-SET-fl/fl^ KO peripheral phenotypic observations described above; therefore, the altered gene expression should reflect *Kmt2d* integrin deficits. In unstimulated peripheral CD4^+^ and CD8^+^ bulk control T-cells, *Itgb2*, *Itgb7*, and *Itgal* were highly expressed, but upon stimulation, the expression was downregulated (**Figure 5F**). At baseline, unstimulated *Kmt2d*-KO peripheral cells initially had lower levels of *Itgb2* and *Itgb7*, as compared with control cells, but integrins had similarly reduced expression in response to stimulation (**Figure 5F**). *Itgal* was not expressed in either *Kmt2d*-KO peripheral cell population; however, post-activation expression was partially recovered. *Kmt2d*-KO CD4^+^ T-cells completely lacked the expression of *Itgae* and *Itga6*, whereas their CD8^+^ T-cell counterparts showed downregulation of these genes upon activation (**Figure 5F**). Thus, KMT2D regulates integrin expression in both CD4^+^ and CD8^+^ peripheral T-cell populations. Furthermore, when evaluated across naïve stimulated subsets by sc sequencing, CD8^+^ KO T-cells exhibited loss of *Itga4*, *Itgae*, and *Itgb7* expression in the naïve populations that overlap with non-activated cell clusters (clusters 0–3). In contrast, the activated CD8^+^ sc populations (clusters 4 and 5; defined by expressing genes for proliferation or effector function) appear to show lower expression of integrins *Itga4*, *Itgae*, and *Itgb7* in both control and *Kmt2d*-KO cells (**Figure 5G**). Together, these data indicate that select leukocyte-specific integrins are expressed in non-activated peripheral T-cells and are regulated by KMT2D (as seen in thymocytes).

### Shifts in human recent thymic emigrants in individuals with molecularly-confirmed KS1

**Figure 6:**
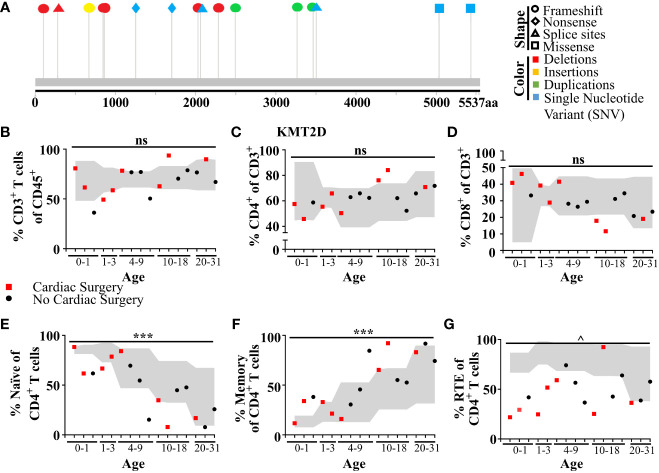
Individuals with KS1 display decreased T-cell counts, naïve CD4^+^ T-cells, and RTE CD4^+^ T-cells, and increased memory CD4^+^ T-cells. **(A)** Individual variants found in KS1 cohort (**Table 1**; n = 16; ages 0–31). Lolliplot indicates protein amino acid location (*via* stick location), types of variant (i.e., shape: squares: missense, circles: frameshifts, triangles: splice sites, diamonds: nonsense), and changes at the nucleotide level (i.e., color: deletions: red, insertions: yellow, duplications: green, and single-nucleotide variant (SNV): blue). **(B-G)** Data from clinical flow cytometry (markers represent separate individuals). **(B)** Percentage of peripheral T CD45^+^CD3^+^ T-cells of total blood cells. **(c)** Percentage of CD4^+^ T-cells **(C)** or CD8^+^ **(D)** of CD3^+^ T-cells. Naive (**E**; CD45RA^+^CD27^+^) or memory CD4^+^ T-cells (**F**; CD45RA^−^) as a percent of CD4^+^ T-cells. Recent thymic emigrants (**G**; RTE; CD27^+^CD45RA^+^CD31^+^) as a percent of CD4^+^ naïve T-cells. **(B-G)** Cardiac surgery (red square; non-surgery: black circle), required for (or performed on) many individuals with KS1, can remove and/or damage the thymus. Values found within gray region represent reference population range (2.5% - 97.5%) for the healthy age-matched individuals. Significance was determined using the binomial test, with the null hypothesis being that individuals with KS1 have a same probability of falling outside the reference range equal to 5%, that is the same as healthy age-matched individuals. The resulting *P*-values were subsequently adjusted for multiple testing with the Bonferroni method and noted by asterisks: *P*-value > 0.05 (ns), *P*-value < 0.001 (***), *P*-value < 1e^−9^ (^), and *P*-value < 1e^−14^ (&). Specifically, in **(B)**, CD3^+^ significance was calculated for values below the standard range only (one-tailed binomial test).

To analyze T lymphocyte disruption in KS1, we recruited a group of individuals (n = 16) with diverse *KMT2D* genetic variants deemed likely pathogenic/pathogenic by the American College of Medical Genetics and Genomics criteria and diagnosed with KS1 by a clinical geneticist (**Figure 6A**, **Table 1**). Upon flow cytometric analysis of peripheral blood cells, we found that CD3^+^ T-cells and CD4^+^ and CD8^+^ T-cell subpopulations were within the age-matched clinical reference ranges (**Figures 6B–D**). Upon further analysis of the CD4^+^/CD8^+^ subpopulations, we observed that individuals with KS1 have a significantly lower percentage of naïve CD4^+^ T-cells and a corresponding expansion of memory CD4^+^ T-cells; however, the corresponding CD8^+^ T-cell compartments were within the clinical reference range (**Figures 6E, F** **,** **Supplementary Figures 7A, B**). Furthermore, individuals with KS1 had a significant reduction in the percentage of CD4^+^CD31^+^ recent thymic emigrants (RTE; **Figure 6G**). Together, these data show that our *Kmt2d* haploinsufficient mouse model partially recapitulates the T-cell phenotype observed in individuals with KS1 (expanded CD4^+^ memory T-cells, reduced RTEs).

**Table 1:** De-identified information collected from 16 individuals with KS1.

SUBJECT#	Age at Evaluation	*Kmt2d* Mutation (NM_003482.4)	Additional VUS
**1**	0 Years, 1 Months	c.9787_9791dupAAGCA	
**2**	0 Years, 4 Months	c.3754C > T	
**3**	0 Years, 5 Months	c.303delG	
**4**	1 Years, 4 Months	c.10394dupG	
**5**	2 Years, 1 Months	c.5104C > T	
**6**	4 Years, 10 Months	c.16295G > A	
**7**	5 Years, 8 Months	c.2533delC	
**8**	7 Years, 2 Months	c.6086delC	
**9**	7 Years, 4 Months	c.839 + 1delG (Aka: IVS7 + 1delG)	VUS c.5867+14C > T, n/a VUS c.6998C > T; p.P2333L
**10**	12 Years, 6 Months	c.2578_2579delCT	
**11**	15 Years, 5 Months	c.15104G > C	
**12**	17 Years, 0 Months	c.7481dupT	
**13**	17 Years, 10 Months	c.10507 + 2 T > G (Aka: IVS37 + 2 T > G)	
**14**	23 Years, 9 Months	c.6183 + 3 G > T (Aka: IVS29 + 3 G > T)	
**15**	24 Years, 10 Months	c.2008_2009insT	
**16**	30 Years, 7 Months	c.6844delC	

De-identified variant information on the KS1 individuals (and the extended version found in **Supplementary Table 6**) corresponding to the data in **Figure 6**/**Supplementary Figure 7**. Each row represents an individual (Column 1). **Figure 6**/**Supplementary Figure 7** graphs are assembled with x-axis in ascending order based off the individuals’ age at the time of lymphocyte evaluation (Column 2). The location/type of the KMT2D variation (insertion [ins], deletion [del], duplication [dup], single-nucleotide variation [>]; Column 3) was further used in to generate data found in **Supplementary Table 6** using VarSome to determine the variation type (missense, nonsense, frameshift, and splice variant), region (within exon/intron), and predict protein amino acid alteration required to generate the **Figure 6** Lolliplot (location of the amino acid change and the type [noted by shape/color]). Known locations of variants of uncertain significance (VUS) for the individuals are indicated (Column 4).

## Discussion

Adhesion is vital for immune responses, influencing development, maturation, homing to tissues, and signaling for function. Dysregulation of integrins are associated with compromised immunity or cancer (reviewed in [50, 55–59]). Herein, we have found that KMT2D, an epigenetic regulator, plays a key role in T-cell development by regulating integrin expression, affecting cell fate, location, and function. In *Kmt2d*-deficient mice, crucial leukocyte integrins (*Itga4*, *Itgae*, *Itgal*, *Itgb2*, *Itgb7*) are dysregulated and signaling is altered, impacting maturation and activation.

The specific timing of dynamic integrin “switches” (shifts in integrin heterodimers composition) have major impacts on both thymocyte development and peripheral T-cell function by controlling migration (adhesion, motility), activation, and/or maturation capabilities. For example, ITGA4-ITGB1 [VLA4] and ITGA5-ITGB1 [VLA5] heterodimers are reported to mediate motility in mature CD3^hi^CD69^hi^ thymocytes, whereas T-cells require expression of ITGAL–ITGB2 [LFA1] alone or in combination with VLA4 for adhesion (60, 61). ITGB2 activation influences ITGB1-specific adhesion capacity and fate decisions. The relative proportion/co-expression of heterodimers can determine cell adhesion or motility, thus influencing fate (60–62). We observed specific integrin expression switches (downregulation of ITGAL/*Itgal* and ITGB2/*Itgb2* and upregulation of *Itga5* and *Itgb1*), which may significantly alter the capacity of the *Kmt2d*-deficient thymocytes/T-cells to interact and/or correctly localize to receive necessary signals for selection, maturation (licensing), or activation. We further demonstrated the impaired contractile-based migration of *Kmt2d-*deficient cells toward chemokines, thus suggesting that the defect is linked to lack of LFA1. Another integrin pair, ITGAE-ITGB7, binds SP to medullary zone epithelial cells for required localization of SP thymocytes during maturation and selection (63). Without *Kmt2d*, both ITGAE and ITGB7 are downregulated in SP thymocytes, likely driving the excessive thymic accumulation of CD8^+^SP cells in *Kmt2d* KO animals. M1-SP accumulation and population level reduction in SP licensing genes in *Kmt2d*-KO cells suggest that KMT2D has an important role in SP maturation. The apparent KMT2D-driven switching from VLA5 toward leukocyte-specific integrins (ITGAE-ITGB7 and LFA1) may be a critical step for proper development of SP thymocytes.

Similarly, peripheral T-cell migration also requires various integrin combinations, either exclusive or promiscuous use of LFA1, VLA4, or LPAM1 (64–68). These integrin heterodimers facilitate cell extravasation from blood, whereas other integrins, such as ITGAE-ITGB7, mediate cell retention within tissues (69). Therefore, the expression of integrins, as regulated by KMT2D activity, may significantly impact peripheral T-cell localization and function. Furthermore, Peyer’s patches (the immune localization of lymphocytes in the intestine) are reduced in size and number in KMT2D haploinsufficient mice and in an individual with KS1; however, it is not clear if this is caused by peripheral lymphocyte migration defects or more likely attributed to organogenesis defects (2).

While we did not observe any motifs of a putative KMT2D transcriptional regulator partner, other investigators have demonstrated a protein C-ets-1 site, retinoic acid (RA) receptor (RAR) site, and a high-mobility group (HMG) binding site within both *Itgb7* and *Itga4* promoter regions. Furthermore, retinoic acid can rescue some gene expression changes found in *Kmt2d*-deficient cells (70–73). In peripheral T-cells, RA has been shown to be required for *Itga4* upregulation and enhanced *Itgb7* expression, but not for *Itgb1 (* 74 *).* Interestingly, the ITGB1 concentration regulates ITGA4-ITGB7 in CD4^+^ T-cells, as ITGA4 preferentially pairs with ITGB1 and only pairs with ITGB7 when in surplus, but the ITGB7 level does not inversely influence ITGB1 concentration (75). We observed a shift toward *Itgb1* and away from *Itgb7* in our peripheral *Kmt2d*-deficient T-cells. Furthermore, we noted the loss of *Itgal* and *Itgb2* in our bulk *Kmt2d*-deficient cells. Their altered expression has been described to impact peripheral CD4^+^ T-cell expression of LPAM1 and CCR9 and downstream homing to mucosal sites (76). In addition to potentially indirect ITGB1- or LFA1-activated compensatory mechanisms, our H3K4 ChIP analyses suggest that *Itgb7* is directly regulated by KMT2D through *cis*-regulatory regions; thus, there may be several contributing factors which reduce ITGB7 expression in *Kmt2d*-KO cells. With defective ITGB7/LPAM1 peripheral lymphocyte mucosa-homing in *Kmt2d*-deficient models (2), localization may be a shared KMT2D-driven adaptive immune deficit in individuals with KS1 resulting in compromised mucosal immunity.

LFA1/ITGAE-ITGB7-VLA5 integrin switches caused by the loss of KMT2D may have substantial implications in downstream adhesion capacity and fate decision making (i.e., SP maturation). How these shifts influence thymocyte/T-cell activation is likely complex, altering multiple important lymphocyte cellular processes. One major implication of KMT2D-driven integrin switching may involve the immunologic synapse where LFA1:ICAM interactions occur between the T-cells and antigen-presenting cells (APC). LFA1 is recruited as part of the peripheral supramolecular activation cluster (pSMAC) and provides important proximal TCR stimulation/costimulatory function through facilitating TCR accumulation, CD45 removal from the synapse, formation of stable adhesion with the APC through ligand interactions with ICAM, and co-engagement signaling needed for activation (77, 78). Normally, the LFA1/ICAM interaction lowers the optimal T-cell stimulation threshold to assist in downstream signaling of extracellular signal–regulated kinase (ERK) 1/2 during T-cell activation (79). In CD8^+^ T-cells, TCR signaling activates ERK1/2 downstream signaling, which recruits LFA1 to enhance adhesion. In a feedback loop, LFA1 downstream signaling, then further sustains ERK1/2 separately through cytohesin-1 for “optimal” T-cell activation (78). Therefore, cells with reduced LFA1 would require a higher TCR stimulation threshold, thus impacting both thymic selection and peripheral immune responses. Interestingly, KMT2D has been previously shown to regulate both RAP1A and RAP1B, which are small, TCR-inducible GTPases that regulate downstream RAS-mediated activation of ERK-driven fate decisions (e.g., cell cycle, differentiation, growth) (80, 81). In KS1 lymphoblasts, the super-enhancer (defined by both H3K27ac/H3K4me1/2 histone modifications; also known as an “active” enhancer) of RAP1A displayed a significantly reduced H3K4me1/2 signal, thus demonstrating a direct impact on another gene associated with T-cell activation (82). Overall, T-cell activation signaling may be regulated by KMT2D at several overlapping levels and via simultaneous independent pathways.

The impact of SP thymocyte TCR activation deficiencies (such as downstream abnormally sustained high levels of CD69 or deficiency in CD69-repressed egress-licensing genes [*Foxo1* or *Klf2*] *(* 47, 83–85)) is that without proper licensure signaling, there is a failure to egress, resulting in decreased naïve T-cell counts. Based on our findings in *Kmt2d* KO models, we now hypothesize that the M1-SP accumulation occurs due to suboptimal SP thymocyte TCR signaling during selection (also partially supported by the observations made by Placek *et al*. regarding a reduction in thymic regulatory cells (13), which occurs as part of the M1–M2 transition), thus impacting downstream CD69/FOXO1 “licensing” signaling, which subsequently blocks thymic egress. Interestingly, the presence of thymic hypertrophy, which differed between the two T-cell–specific *Kmt2d* KO models (whereas no observable hypertrophy was observed in the haploinsufficient model), might be associated with the level of blocked egress. Although the precise mechanisms remain unclear at this time, a possible explanation may relate to the timing of the Cre recombinase expression, which differs slightly between models (with *Lck*-Cre^Mar^ turning on at DN3 compared with DN4 for *CD4*-Cre) (22). Thymic egress is supported by our observation of T-cell lymphopenia and by the near-complete absence of KMT2D locus recombined cells in the peripheral T-cells of *Kmt2d* KO (*Lck* ^Mar^-Cre^+^ *Kmt2d* ^-SET-fl/fl^) mice. Furthermore, the difference observed in peripheral T-cell numbers between *Kmt2d* KO and *Kmt2d* haploinsufficient models may be mediated by KMT2D dosage, with one functional allele being sufficient to permit egress. However, the *Kmt2d* haploinsufficient model does not seem to share M1 enhancement observed in the *Kmt2d* KO, but still displays altered CD8^+^SP accumulation. The specific CD8^+^SP accumulation without a matching CD4^+^SP accumulation, coupled with its permitted egress, suggests that SP subpopulations are potentially independently influenced by the thymic microenvironment or by altered earlier lineage-specific transcription factors, rather than later FOXO1 licensing/S1PR1 egress signaling deficiencies that may bias transition of DP toward CD8^+^SP accumulation. Furthermore, *Kmt2d* haploinsufficient mice additionally display naïve to central memory shifts, thereby suggesting that although the SP is permitted to egress many other mechanistic possibilities may lead to KSAIDs (such as altered cell cycle/proliferation, survival/death, cell–cell contact/signaling, or deviated responses to stimulation via hypoactivation or generation of virtual memory). We also observed the loss of naïve/RTE T-cells in the peripheral T-cell compartment and enhancement of a memory, CD44-expressing, T-cell phenotype in all models and in individuals with KS1. We do not believe that the enhanced peripheral memory cell phenotype in KS1 is caused by lymphopenia-driven homeostatic proliferation, because individuals with KS1 do not have significantly different percentages of total T-cells. However, in *Kmt2d* KO models lymphopenia-driven memory skewing could occur due to their severely reduced overall T-cell counts. A preliminary study described *Kmt2d* KO in CD8^+^ T-cells impacts survival (12), whereas another shows *Kmt2d* KO suppresses IFNγ (11); however, both of these studies were performed in *Kmt2d* KO mice, which we now report Kmt2d KO mice are lymphopenic. As lymphopenia can drive homeostatic proliferation, future studies regarding peripheral T-cells in *Kmt2d* haploinsufficient models would be necessary to understand these shifts removing the confounding influence of lymphopenia.

*Kmt2d* KO models have more severe thymic and peripheral phenotypes as compared with KS1 humans or *Kmt2d* haploinsufficient mouse models, supporting the hypothesis that KMT2D may work in T-cells in a both a dose-related non-redundant manner and equivocally across all deficient models. KS1 is defined as loss of function by the observation of a KMT2D/KMT2D human variant displaying the KS1 phenotype with equal severity to that of a KMT2D/+ KS1 individuals (86). In contrast, *Kmt2d^βgeo/βgeo^* mice are embryonically lethal. The addition of the artificial cassette (*βGeo*) utilized to induce truncating protein may contribute to unexpected impact and thus may explain lethality compared with viability of the KMT2D/KMT2D variant. Overall chromatin modifiers, including KMT2D, other histone methyltransferases, such as DOT1L, and *Kdm6a*, the gene encoding a lysine demethylase and haploinsufficient in individuals with KS2, can clearly impact lymphocyte differentiation through modulating the development of thymocytes and by controlling naïve versus memory composition of peripheral T-cells (87, 88). We have assessed overlapping gene changes between *Kmt2d* KO with *Dot1l* KO CD8^+^SP thymocytes (GEO accession: GSE138910) and found that only three genes were shared despite similar phenotypes. Therefore, further investigation of how epigenetic regulation sculpts T-cell development may inspire new approaches to modify immune function.

In summary, we show the use of *Kmt2d* multi-model systems strengthen the knowledge of consistent valid *Kmt2d*-driven KSAID phenotypes, such as *Kmt2d* loss in both humans and murine KS1 models led to specific thymic and T-cell abnormalities. KMT2D has a direct intrinsic effect within thymocytes on integrin expression, which controls activation and adhesion, and dosage-related loss (KO only) of SP maturation and licensing gene expression, thus influencing both developmental progression and egress. This aberrant *Kmt2d* KO thymocyte progression leads to downstream peripheral loss of T-cells, whereas across all models, we observe a reduction of CD4^+^ RTEs and a skewing toward a memory T-cell phenotype. These peripheral changes are likely to mediate major aspects of KSAID and should be the focus of further clinical research.

## Data availability statement

The RNA-seq data discussed in this publication have been deposited in NCBI's Gene Expression Omnibus (GEO) (89) and are accessible through GEO Series (GSE) accession GSE205285. Additionally, the publicly available datasets from GSE accessions: *Kmt2d*-KO and control datasets (GSE69162, GSE204946, GSE217656 and NCBI SRA PRJNA541991) and *Dot1l*-KO and control dataset (GSE138910) were included in our analyses and are linked to GSE205285.

## Ethics statement

The studies involving humans were approved by Cincinnati Children’s Hospital Medical Center [CCHMC] Institutional Review Board [IRB]. The studies were conducted in accordance with the local legislation and institutional requirements. Written informed consent for participation in this study was provided by the participants’ legal guardians. The animal study was approved by Institutional Animal Care and Use Committee of Cincinnati Children’s Hospital Medical Center [CCHMC] and Johns Hopkins University [JHU]. The study was conducted in accordance with the local legislation and institutional requirements.

## Author contributions

SP: Conceptualization, Data curation, Formal analysis, Investigation, Methodology, Project administration, Supervision, Validation, Writing – original draft, Writing – review & editing. LZ: Conceptualization, Funding acquisition, Investigation, Methodology, Project administration, Resources, Supervision, Writing – review & editing. MK: Formal analysis, Software, Validation, Writing – review & editing. YW: Investigation, Writing – review & editing. CS: Investigation, Writing – review & editing. KS: Formal analysis, Writing – review & editing. LB: Formal analysis, Writing – review & editing. DQ: Data curation, Writing – review & editing. OB: Data curation, Writing – review & editing. BS: Data curation, Writing – review & editing. AB: Funding acquisition, Project administration, Resources, Supervision, Writing – review & editing. AL: Conceptualization, Funding acquisition, Investigation, Methodology, Project administration, Resources, Supervision, Writing – original draft, Writing – review & editing. HB: Conceptualization, Funding acquisition, Investigation, Methodology, Project administration, Resources, Supervision, Writing – original draft, Writing – review & editing.
