# Supplementary material for: KMT2D regulates activation, localization, and integrin expression by T-cells
Source: Front Immunol. 2024 May 3;15:1341745. doi: 10.3389/fimmu.2024.1341745 (PMC11099208; doi:10.3389/fimmu.2024.1341745)
Supplement: Supplementary file 1 [file DataSheet_1.docx]

**Supplementary Table 1: Flow cytometry panels**

| **Antibody** | **Fluorophore** | **Company** | **Catalogue #** | **Clone** | **Panel** |
| --- | --- | --- | --- | --- | --- |
| CD3e | PE-Cy5 | Biolegend | 100310 | 145-2C11 | † Peripheral |
| CD4 | eFluor650 | Biolegend | 100469 | GK1.5 | † Peripheral |
| CD8 | eFluor605 | Biolegend | 100744 | 53-6.7 | † Peripheral |
| CD44 | AF488 | Biolegend | 103016 | IM7 | † Peripheral |
| CD62L | APC-Cy7 | Biolegend | 104428 | MEL14 | † Peripheral |
| Live/Dead (Blue-fluorescent reactive dye) | ZombieUV | Invitrogen/  Thermofisher Sci. | L23105A | n/a | † Peripheral/ ‡ §Semimature/Integrin/ ¶ Thymus Population |
| CD69 | PE | Biolegend | 104507 | H1.2F3 | ‡ Semimature |
| CCR7 | PerCP (Cy5.5) | Biolegend | 120115 | 4B12 | ‡ Semimature |
| MHCI | APC | eBioscience/Invitrogen/  Thermofisher Sci. | 17-5958-82 | AF6-88.5.5.3 | ‡ Semimature |
| TCRbeta | FITC | Biolegend | 109205 | H57-597 | ‡ Semimature |
| CD4 | PE-Cy7 | BD Bioscience | 561099 | Rm4-5 | ‡ Semimature |
| CD8 | V450 | BD Bioscience | 560471 | 53-6.7 | ‡ Semimature |
| CD4 | BV650 | Biolegend | 100469 | GK1.5 | §Semimature/Integrin |
| CD8 | BV605 | Biolegend | 100744 | 53-6.7 | §Semimature/Integrin |
| CD24 | PacificBlue | Biolegend | 101820 | M1/69 | §Semimature/Integrin |
| TCRbeta | BUV737 | Biolegend | 612821 | H57-597 | §Semimature/Integrin |
| ITGAL | APC | Biolegend | 101120 | M17/4 | §Semimature/Integrin |
| ITGAE | PerCP-eF710 | eBioscience/Invitrogen/  Thermofisher Sci. | 46-1031-82 | 2E7 | §Semimature/Integrin |
| ITGB7 | APC | Biolegend | 321207 | FIB504 | §Semimature/Integrin |
| CD4 | BV650 | Biolegend | 100469 | GK1.5 | ¶ Thymus Population |
| CD8 | BV605 | Biolegend | 100744 | 53-6.7 | ¶ Thymus Population |
| CD25 | PE-Cy7 | Biolegend | 102016 | PC61 | ¶ Thymus Population |
| CD44 | AF488 | Biolegend | 103016 | IM7 | ¶ Thymus Population |
| TCRbeta | BUV737 | Biolegend | 612821 | H57-597 | ¶ Thymus Population |

† Peripheral : *Kmt2d*^+/βgeo^ and *CD4-*Cre; *Kmt2d*^SET-fl/fl^ mouse lines.

‡ Semimature : *Lck-*Cre^Mar^; *Kmt2d*^SET-fl/fl^ mouse line.

§ Semimature/Integrin : *Kmt2d*^+/βgeo^ and *CD4-*Cre; *Kmt2d*^SET-fl/fl^ mouse lines.

¶ Thymus Population : *Kmt2d*^+/βgeo^ and *CD4-*Cre/Vav1-iCre; *Kmt2d*^SET-fl/fl^ mouse lines.

**Supplementary Table 2: q-PCR Recombination Primers**

| **Primer Forward/**  **Reverse Name** | **Primer Sequence** | **Expressed** |
| --- | --- | --- |
| *Kmt2d*-Exon50 -For | 5'-CTG TGT GGA ACC GCA TCA TTG -3' | Control Expressed  KO Not Expressed |
| *Kmt2d*-Exon50 -Rev | 5'-CAG CCC AAA GAG TTC TTC GCC -3' |  |
| *Kmt2d*-Total R2 -For | 5'-TCT CCC GAG ACT CAG TCA CTG-3' | Control Expressed  KO Expressed |
| *Kmt2d*-Total R2 -Rev | 5'-CAG TTG AGC TAG TCA AGT GAT T -3' |  |
| *Kmt2d-*P3e-For | 5'-TTC CAT AGC CAT TGC TCA AA -3' | Control Not Expressed  KO Expressed |
| *Kmt2d-*F1r-Rev | 5'-GAA CGG ATC CAA GCT TAT GC -3' |  |
| *Kmt2d-*F1-For | 5'-GCA TAA GCT TGG ATC CGT TC -3' | Control Expressed  KO Expressed |
| *Kmt2d-*R2-Rev | 5'-CTG AAG TTT GGG AGG GTC AC -3' |  |

**Supplementary Table 3: ChIP-PCR Primers**

| **Primer** | **Strand** | **Sequence** | **Location of Seq in mm10 (UCSC In-Silico PCR)** |
| --- | --- | --- | --- |
| ***Klf2* Set 1** | *Sense* | GCCTATCTTGCCGTCCTTT | chr8:72319092+72319237 146bp |
|  | *Anti-Sense* | TGGACCTTGTCATCTCCAGTA |  |
| ***Klf2* Set 2** | *Sense* | CTTGAGGGCCTAGTTGTTAGAC | chr8:72319344+72319453 110bp |
|  | *Anti-Sense* | CCGCCTCGGGTTCATTT |  |
| ***Itgb7* Set 1** | *Sense* | CTCAGGGTCTTCAAGGTTACAG | chr15:102225073+102225193 121bp |
|  | *Anti-Sense* | GTCGTCTTTATCGTCCTCCTTC |  |
| ***Itgb7* Set 2** | *Sense* | CTGGGAGAAGCACAAGAGTAAG | chr15:102231561+102231678 118bp |
|  | *Anti-Sense* | CTCCTGCTGCTCATCCATTT |  |
| ***ItgaL* Set 1** | *Sense* | TCCAGCTGTTTGGTAGGAAATC | chr7:127296512+127296615 104bp |
|  | *Anti-Sense* | GAGTGGCTCCTCATCTTCTTTG |  |
| ***ItgaL* Set 2** | *Sense* | AGTGAGCCTTCACGTGTTTAG | chr7:127296735+127296819 85bp |
|  | *Anti-Sense* | CAAAGGAGGTGGAGTAGAAACC |  |
| ***Gapdh*** | *Sense* | CTC CTG CGG CCC ACT CCG CGA | chr6:125165524+125165626 103bp |
|  | *Anti-Sense* | CGG ACTGCA GCC CTC CCT GGT |  |

**Supplementary Table 4: Top genes in Topp-Fun analyses GO: Biological processes categories from SP *Kmt2d* KO downregulated gene list.**

- Activation: Overlapping genes found in terms: (1) cell activation; (2) T cell activation; (5) lymphocyte activation; (6) regulation of cell activation; (9) leukocyte activation; (11) regulation of T cell activation; (13) regulation of leukocyte activation; (14) regulation of lymphocyte activation.
- Adhesion: Overlapping genes found in terms: (4) cell adhesion; (8) cell-cell adhesion; (10) regulation of cell adhesion; (15) leukocyte cell-cell adhesion.
- GTPase: (17) small GTPase mediated signal transduction.
- Other: (3) regulation of immune system process; (7) localization within membrane; (12) protein localization to membrane; (16) negative regulation of immune system process.

| **Activation genes:** | **GTPase genes:** | **Adhesion genes:** |
| --- | --- | --- |
| IL12RB1 | SH2D3C | IL12RB1 |
| TNFAIP8L2 | SH3BP1 | TNFAIP8L2 |
| TNFRSF9 | DGKZ | RARA |
| RARA | ARHGAP9 | ERF |
| IMPDH1 | RRAD | HLA-DOA |
| HLA-DOA | TIMP2 | ACP5 |
| RPS6KA1 | ARHGEF3 | MYO18A |
| MYO18A | BAIAP2 | FXYD5 |
| NFKBID | PREX1 | AKNA |
| DGKZ | RASAL1 | NFKBID |
| PGLYRP2 | RAC2 | TNFRSF18 |
| ACTN1 | CYTH4 | ACTN1 |
| TSPAN32 |  | TSPAN32 |
| ITGAL |  | ITGAL |
| EMILIN1 |  | EMILIN1 |
| LGALS1 |  | ITGB7 |
| GSN |  | LGALS1 |
| PRR7 |  | GSN |
| ADORA2A |  | STK4 |
| JUND |  | ADORA2A |
| NDRG1 |  | STK10 |
| SLA2 |  | JUP |
| ELF4 |  | PKP3 |
| TNFRSF1B |  | PVR |
| PREX1 |  | BAIAP2 |
| RAC2 |  | PREX1 |
|  |  | RAC2 |

**Supplementary Table 5: Lalign non-identical alignments** **of leukocyte integrins 1kb promoter region**

| **Same Chain** | |
| --- | --- |
| *Itgb2/Itgb7* | 29.5–35.8% |
| *Itgae/Itgal* | 44.6–49.3% |
| **Cross Chain** | |
| *Itgb7/Itgae* | 52.6-56.4% |
| *Itgb7/Itgal* | 0% |
| *Itgb2/Itgae* | 50.6-56.9% |
| *Itgb2/Itgal* | 46-48.1% |

**Example of one of the Itgb2/Itgb7 alignment outputs**

Waterman-Eggert score: 927; 25.5 bits; E(1) < 0.021

35.8% identity (35.8% similar) in 1371 nt overlap (5-986:10-1001)

10 20 30 40 50 60

EMBOSS GCAGAAGCCACATCTCTACCCTGGGGGAATGCACAGACAGCTGCAAAGGCTCCTGAGCCT

::: ::: ::: :::: : :: : : ::: :: :: ::

EMBOSS GCATCAGC---ATC-CTACATTCTGGTATT------ACAAGTGTGAA-----------CT

10 20 30 40

70 80 90 100 110

EMBOSS GATGCCCCCCTCTGCTGGGGTGGGTTGTT--AGCACTGG-------ACAGATGTC-----

::: : :: :::: :: : :: :: :::: :: :

EMBOSS GATACACC----TGCT-----------TTCCAACAATGTTCTTTCAACAGTTGGCATCTT

50 60 70 80 90

120 130 140 150

EMBOSS --------------TCAGGTGTGTGTAGAAGGGGAAATGAGAAGGTGGGTGCTCAGACAT

::: :::: :::: :: :: ::::

EMBOSS TTGCACATTTTTTTTCAACTGTG---AGAA---GATAT-AGAA-----------------

100 110 120

160 170 180 190 200

EMBOSS CAGAATGTGGATGTGGGTTTGGTAGGGAGAGTAATACCGGTCCTT---------------

::: :: :: :::: :::: : ::: :::

EMBOSS ---AATATGCATAAAGGTTC---AGGGCTATTAA--------CTTCTTGTAACCCTCTCA

130 140 150 160 170

210 220 230 240

EMBOSS -------------GGATGGACTCCTGCACCAGGCCGTCCCATCCCACCCTTGCTGGTGCC

::::: ::::::: : :::: :: :

EMBOSS ACCATTATCACCAGGATG---TCCTGCA---------------------TAGCTGATGTC

180 190 200 210

250 260 270 280 290

EMBOSS TGCCCTTCCCA-TCCGTCCCTGACAGCCT---CTTGTCATTTAGCTACTG---------A

:::: ::: ::::: : :: :::: :: ::: :: :

EMBOSS -------CCCACTCC-------ACAGCTTTATCT--TCATGTAACTA-TGTCTTTAAAAA

220 230 240 250

300 310 320 330 340

EMBOSS TTGTACTTCATGGCTTGACATA------GAGGGTTAGAGA-AGTGGGGTGACCACACTTT

:: ::: :: : :::: :: ::: : ::: : :::: :

EMBOSS TTCTAC--CAAG------CATACTACACGA-----AGAAAGAGT------AACACA---T

260 270 280 290

350 360 370 380

EMBOSS GAGCCCTCTCCCCAGGTTCCTTCTGCC------------------CCT--GCTGTGGCC-

:: : ::::::: :::: ::: ::: ::::::: :

EMBOSS GAAC-----ACCCAGGT--CTTCAGCCAACACCCCCCCCCCCAACCCTCCGCTGTGGTCA

300 310 320 330 340

390 400 410

EMBOSS ----CTGGGCTTT------TATC----------TTCAATTCCCCTCCTA-----------

:::: :: : :::: : ::: ::: ::::

EMBOSS CCTCCTGGCCTCTGTCTTGTATCCACACCCACCTCCAACCCCCATCCTGATTACAAGGAA

350 360 370 380 390 400

420 430 440

EMBOSS ---------CCGTGCT------CCATTT-------TTT-----------TTTAAATATAC

:: :: : :::::: ::: : :::: :: :

EMBOSS GAAAATCTGCCATGTTTTTCTACCATTTGCGAGCATTTCAGCATGTAACTCTAAAGATGC

410 420 430 440 450 460

450 460 470 480

EMBOSS TTATTTTGCTATGGGGGAGGGG-----AACTATCAAAT--------GGACAAGATT--AC

:: :: :: ::: :: ::: ::: :::: : ::

EMBOSS A-------------GGTAGTGGGATGTAACCATGAAACATTGTTGCGGA-AAGAATTAAC

470 480 490 500 510

490 500 510 520

EMBOSS TTTTAATGTAGTTTTT--ATAAG-------------ATGTTATA------------AACT

: :::: : : ::: :: :::: ::::

EMBOSS T---------GTTTCTCAACAAGGCTGACATCTCAAATCTTATGCTAAAGGTTCCCAACT

520 530 540 550 560

530 540 550

EMBOSS --------TGTATCTTTTT--------------------ACCATTAAAGTGCAGTGTATG

::: ::: ::: :: : : : ::: :: : ::

EMBOSS CCCTTTGGTGTCTCTCTTTCTGCCCTCTCTCCTCTTGCAACTAGTCAGGTGGGGT-TTTG

570 580 590 600 610 620

560 570 580 590

EMBOSS TTC--------CTTCGTGACATATTTAG----------GATATTTAAATAAAAAGGATGG

::: ::: :::: :: :: : :: ::::

EMBOSS TTCACCACTAACTTTGTGA----TTGAGCTCCTACAGTGTTACTTAAC------------

630 640 650 660

600 610 620 630 640

EMBOSS CGCTTTTCTACAT---TCCATGTCC---TCTTTGAGAAGACTTGATTTGTTGGGGAAGC-

:::: :::::: :: ::: ::

EMBOSS ---------ACATCCCTCCATGCCCCACTCT--------------------------GCC

670 680 690

650 660

EMBOSS ---TTTTTCTCCAAAGG----------------------CCA---------------GAG

::: ::: : ::: ::: :::

EMBOSS CCATTTCTCTGCCCAGGACCTTCCCCTGCCTCCTTCTTCCCATTCGCCTGCTTCCTCGAG

700 710 720 730 740 750

670 680 690 700 710 720

EMBOSS TGGTCT--CAGTGGGGTGGTTTTCCTCCCTCAGGACCCCA-CGGCTTGCAGCAGAGTCGA

::: :: :: :::::: :::: : :::: ::: : :::

EMBOSS TGGGCTACCA-----------------CCTCAG---CCCATCCACTTGTAGCTGGGTC--

760 770 780

730 740 750 760

EMBOSS CGGGCCCCGTTAAGACTTCTTTTCTCCAGA--GGGAAGC------ATCAG----------

::::: ::::: : ::: : :::::

EMBOSS ---------------CTTCTC----CCAGACTGAGAACCCACACAATCAGCCTCTGCTTC

790 800 810 820

770 780

EMBOSS ---------GT--------------------------------AGAGGCAAAAGTCATTG

:: :::: : : : :

EMBOSS CGCCTGCTGGTCCATGAATCCCTACCTCTTCTTGACATGTGAGAGAGCC------CCTCG

830 840 850 860 870 880

790 800 810 820 830 840

EMBOSS ATGCTAAAAAAAAAAAAAAAAAAAAAAAAAAAAGGGAGGGGACTCTCAACAGTCAGACAT

:::::: ::::

EMBOSS ATGCTA---------------------------------------TCAA-----------

890

850 860 870 880

EMBOSS AATCAGGGCTGACTCCTGCTAGGCAGGGTCC------------------CTT--CG---A

:: :: ::::: ::: ::: :: :

EMBOSS ---------TGGCTGCTGCT--------TCCAACAGTAGCCCCTCCACTCTTGCCGTAAA

900 910 920 930

890 900 910 920 930 940

EMBOSS AGGTGGTGCCTCTGGCTTCCAAGGCTCTGCCACTGGCTCAGGTGAGAGGCCTACATAGTC

:: : :: : :: :: : :::: : :: :: :: :::::

EMBOSS AGCTTGT-CATC--GCCTACAAGCCCCTCCCTCT--CTCAG-------------------

940 950 960 970

950 960 970 980

EMBOSS TGGTTTGTTGTTTGTTTCTGGTTTT-----GTCCCGGAGTGAA---GTAAA

::: ::: : : ::::: : :::::

EMBOSS ------------TGTCTCT---TCTCTAACGTCCC-------ACGTGTAAA

980 990 1000

**Supplementary Table 6: De-identified information collected from 16 individuals with KS1.**

Data from this information corresponds to **Figure 6/Supplementary Figure 7.** Each column represents an individual. **Figure 6** graphs are assembled in ascending order (Row 1) based off the individuals’ age at the time of lymphocyte evaluation (Row 2). The following information are provided: location of the KMT2D variation and the type of change (single-nucleotide variation, duplication, insertion, deletion, etc; Row 3); information, such as start position (Row 4) and end position (Row 5) as located on the chromosome 12, nucleotide(s) of the reference genome (Row 6) and what the variation changes in the individual (Row 7) were required to generate the lolliplot (Rows 4-7); VarSome determination of the variation type (missense, nonsense, frameshift, and splice variant; Row 8); location where the variation takes place (within what *KMT2D* exon/intron; Row 9); VarSome KMT2D protein amino acid prediction (Row 10); location of the amino acid change on the lolliplot (Row 11) and the type (noted by shape/color; Row 12); and known locations of variants of uncertain significance (VUS) for the individual are indicated (Rows 13-15). Abbreviations found in Row 3: insertion (ins), deletion (del), duplication (dup), single nucleotide change (>). Complete information of the abridged version found in **Table 1**.

| **16** | **15** | **14** | **13** | **12** | **11** | **10** | **9** | **8** | **7** | **6** | **5** | **4** | **3** | **2** | **1** | **SUBJECT NUMBER** |
| --- | --- | --- | --- | --- | --- | --- | --- | --- | --- | --- | --- | --- | --- | --- | --- | --- |
| 30 Years, 7 Months | 24 Years, 10 Months | 23 Years, 9 Months | 17 Years, 10 Months | 17 Years, 0 Months | 15 Years, 5 Months | 12 Years, 6 Months | 7 Years, 4 Months | 7 Years, 2 Months | 5 Years, 8 Months | 4 Years, 10 Months | 2 Years, 1 Months | 1 Years, 4 Months | 0 Years, 5 Months | 0 Years, 4 Months | 0 Years, 1 Months | **Age at Evaluation** |
| c.6844delC | c.2008_2009insT | c.6183 + 3 G > T  (Aka: IVS29 + 3 G > T) | c.10507 + 2 T > G (Aka: IVS37 + 2 T > G) | c.7481dupT | c.15104G > C | c.2578_2579delCT | c.839 + 1delG  (Aka: IVS7 + 1delG) | c.6086delC | c.2533delC | c.16295G > A | c.5104C > T | c.10394dupG | c.303delG | c.3754C > T | c.9787_9791dupAAGCA | ***Kmt2d* Mutation (NM_003482.4)** |
| 49040926 | 49051675 | 49041915 | 49034410 | 49040289 | 49026862 | 49051104 | 49053476 | 49042112 | 49051150 | 49022633 | 49044284 | 49034628 | 49054625 | 49049834 | 49037565 | **Start_Position** |
| 49040926 | 49051675 | 49041915 | 49034410 | 49040289 | 49026862 | 49051105 | 49053476 | 49042112 | 49051150 | 49022633 | 49044284 | 49034628 | 49054625 | 49049834 | 49037569 | **End_Position** |
| C | - | G | T | T | G | CT | G | C | C | G | C | G | G | C | AAGCA | **Nucleotide(s) Original** |
| - | T | T | G | TT | C | - | - | - | - | A | T | GG | - | T | AAGCAAAGCA | **Nucleotide(s) Changed** |
| Frameshift | Frameshift | Splice Variant | Splicing Variant | Frameshift | Missense | Frameshift | Splicing Variant | Frameshift | Frameshift | Missense | Nonsense | Frameshift | Frameshift | Nonsense | Frameshift | **Mutation Type** |
| Exon 32 | Exon 11 | Intron 30 | Intron 38 | Exon 32 | Exon 49 | Exon 11 | Intron 7 | Exon 29 | Exon 11 | Exon 52 | Exon 22 | Exon 37 | Exon 4 | Exon 12 | Exon 35 | **Location Exon / Intron** |
| R2282Gfs*4 (p.Arg2282GlyfsTer4) | P670Lfs*7 (p.Pro670LeufsTer7) | n/a | n/a | A2496Sfs*10 (p.Ala2496SerfsTer10) | C5035S (p.Cys5035Ser) | L860Vfs*5 (p.Leu860ValfsTer5) | n/a | P2029Lfs*18 (p.Pro2029LeufsTer18) | R845Gfs*85 (p.Arg845GlyfsTer85) | R5432Q (p.Arg5432Gln) | R1702* (p.Arg1702Ter) | P3466Tfs*2 (p.Pro3466ThrfsTer2) | S102Afs*28 (p.Ser102AlafsTer28) | R1252* (p.Arg1252Ter) | Q3265Sfs*67 (p.Gln3265SerfsTer67) | **KMT2D Protein / Intron** |
| 10 | 3 | 9 | 14 | 11 | 15 | 5 | 2 | 8 | 4 | 16 | 7 | 13 | 1 | 6 | 12 | **# of Mutation in Lolliplot** |
| Red Circle | Yellow Circle | Blue Triangle | Blue Triangle | Green Circle | Blue Square | Red Circle | Red Triangle | Red Circle | Red Circle | Blue Square | Blue Diamond | Green Circle | Red Circle | Blue Diamond | Green circle | **Mutation Type in Lolliplot** |
|  |  |  |  |  |  |  | VUS c.5867+14C > T, n/a  VUS c.6998C > T; p.P2333L VUS c.8001A > G; p.P2667P |  |  |  |  |  |  |  |  | **Additional VUS** |


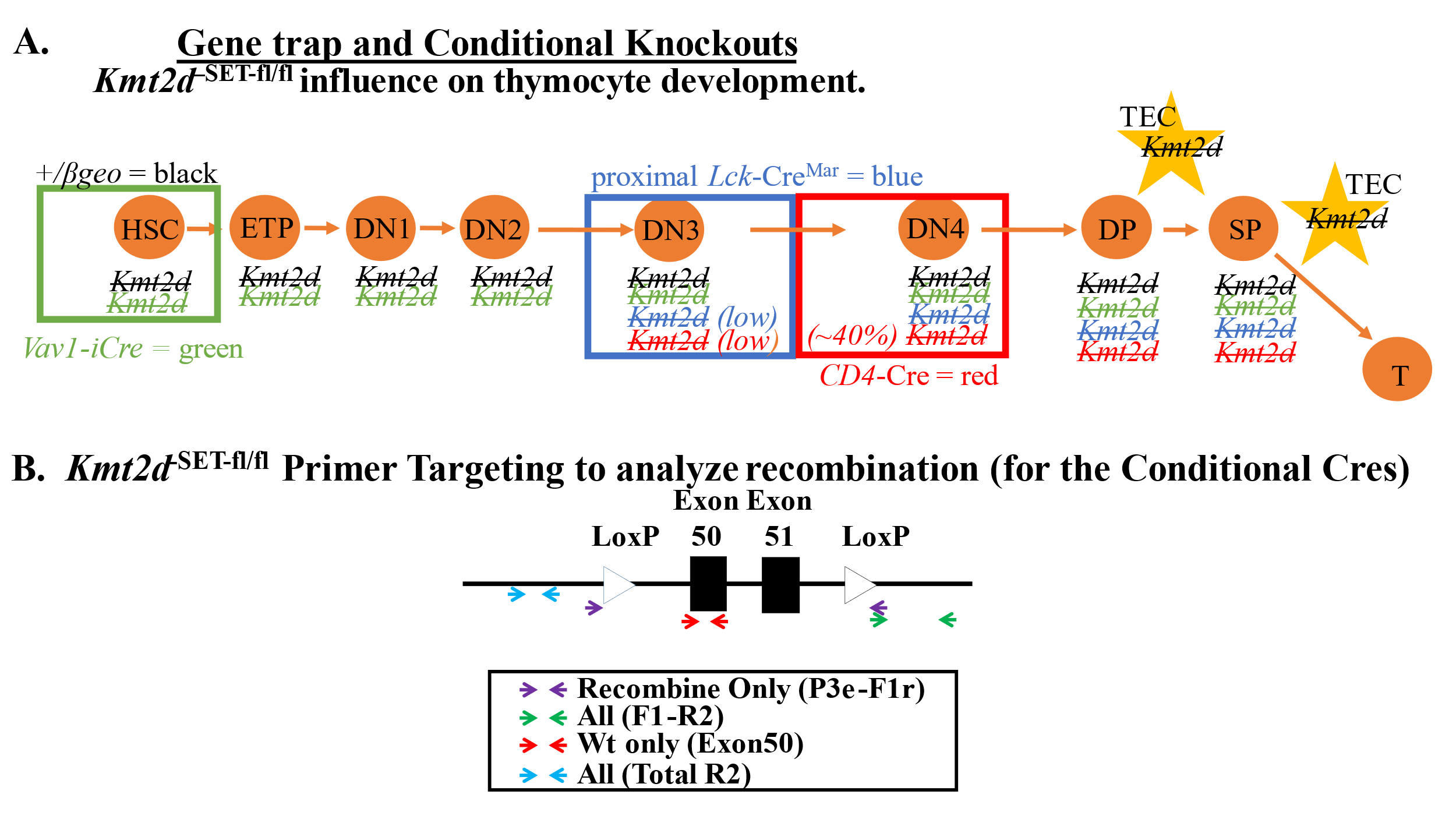


**Supplementary Figure 1. Differentially timed murine constitutional and conditional models *of Kmt2d* loss during thymocyte development.** (**A**) Conditional Cre systems, when specific Cre recombinases turn on according to literature during thymocyte development depicted through loss of *Kmt2d* (shown via strike-through). Thymocytes in orange, supporting cells in yellow. Cres resulting in *Kmt2d KO*: *Vav1*-iCre (green), *Lck*-Cre^Mar^ (blue), *CD4*-Cre (red). Additionally, constitutive *Kmt2d*^+/^*^βgeo^* mouse line shown in black (in both thymocytes and supporting cells). (**B**) Conditional mice harbor *LoxP* sites around *Kmt2d* exons 50 and 51 and in presence of Cre disrupt the SET domain. Analysis of recombination qPCR primers targeted *Kmt2d* SET domain (Determination of recombination: purple (recombine only) / green (all) or the presence of Exon 50: wild-type only (red) / blue (all). See **Supplementary Table 2** for primer sequence information and predicted primer outcomes.

**
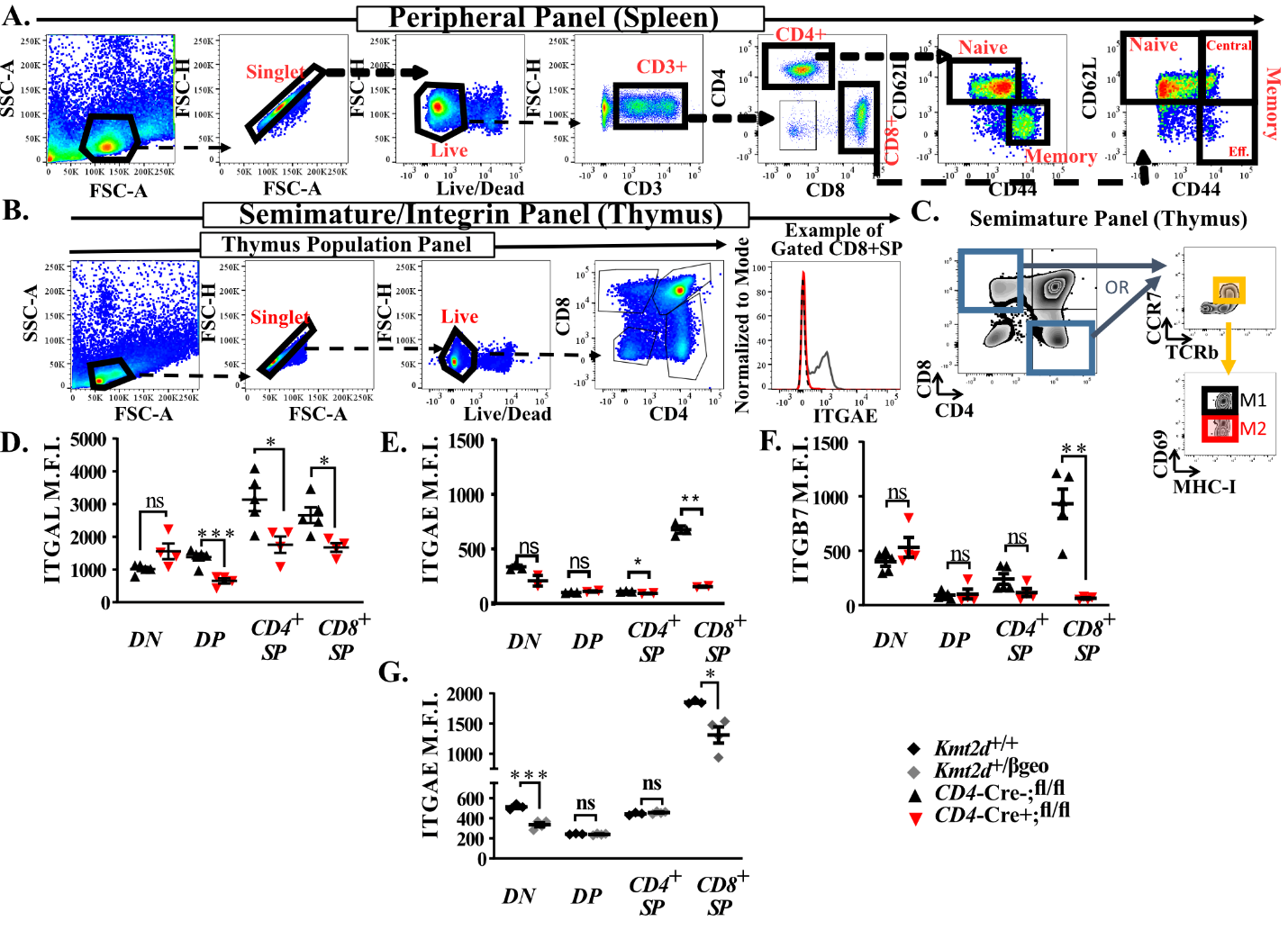
Supplementary Figure 2. Gating Strategy of T cell populations and additional independent experiments of integrin expression.** (**A**) Peripheral Panel (of splenocytes): First, gate on size/granularity (FSC-A vs. SSC-A); on singlets (FSC-A vs. FSC-H); on live (Live/Dead stain negative vs. FSC-H), on CD3 (CD3 positive vs. FSC-H). Next, gate on CD4 by CD8 to separate out the CD4^+^ and CD8^+^ populations. Finally, gate on CD44 by CD62L to separate naïve (CD62L^hi^) versus memory (CD44^hi^) for CD4^+^. For CD8^+^, additionally determination of effector memory (CD62L^lo^) compared to central memory (CD62L^hi^) was defined by expression of CD62L levels on CD44^hi^ cells. (**B-C**) Semimature/Integrin Panel (Thymus). Similar gating strategy of size/granularity, singlet, and live cells as depicted in the peripheral gating (**A**), with subsequent gating on CD4 and CD8 to determine double-negative (DN), double-positive (DP) and single positive (CD4^+^SP, CD8^+^SP) thymocytes. Each population was assessed for integrin expression and intensity (**B**) or SP populations have further gating on on CCR7^hi^ cells (using TCRβ and CCR7; **C**). Lastly, M1 compared to M2 was determined through CD69 expression (as co-gated on MHCI). Alternatively, CD24 with TCRβ can be used in some situations to note M1/M2. (**D**-**F**) 2 additional independent experiments displaying Geometric Mean Fluorescence Intensity (MFI) of ITGAL (**D**), ITGAE (**E**), and ITGB7 (**F**) from *CD4-*Cre; *Kmt2d*^-SET-fl/fl^ or 1 additional independent experiment of ITGAE (**G**) from *Kmt2d*^+/^*^βgeo^* (each mark represents 1 mouse; n = 3 - 5 mice / condition [biological replicates]). Each dataset displays black lines, which represent mean ± SEM. Significance labeled on graphs were determined using a parametric, unpaired, Welch’s corrected *t-test* between a mutant and corresponding littermate controls. Significance based on *P*-value > 0.05 (ns), *P*-value < 0.05 (*), *P*-value < 0.01 (**), *P*-value < 0.001 (***), and *P*-value < 0.0001 (****).


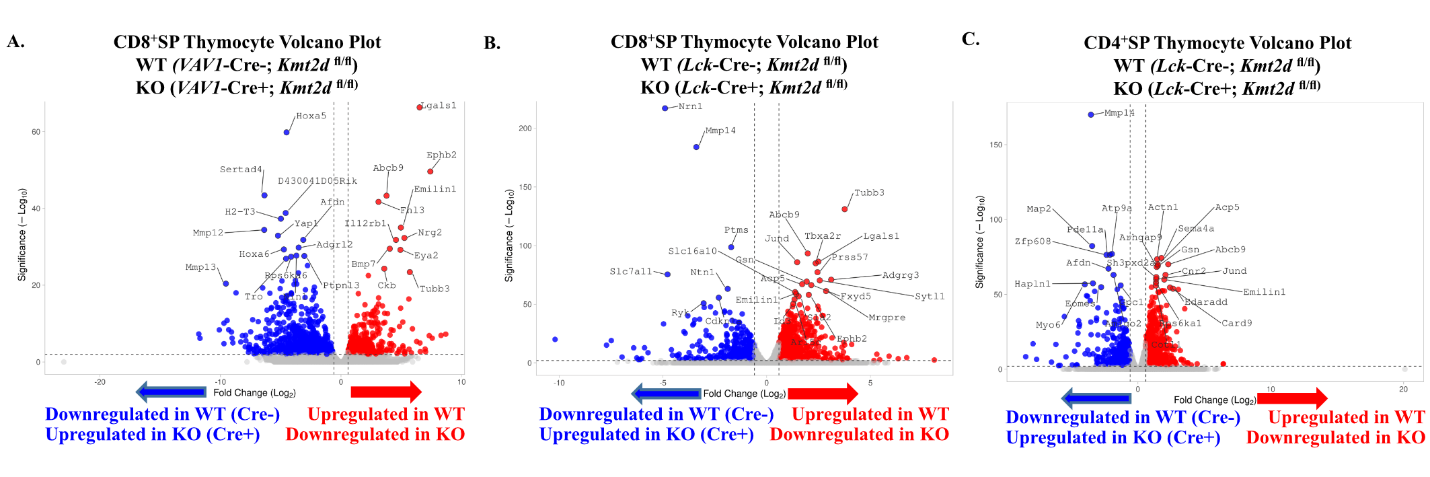


**Supplementary Figure 3. Differential gene expression between SP cells from conditional *Kmt2d*-KO and control littermates.** DESeq2 analyzed CD4^+^SP (**A**) CD8^+^SP (**B**-**C**) RNA-seq from *Lck-*Cre^Mar^ (**A**-**B**) *Vav1*-iCre-driven (**C**) *Kmt2d*-KO and control littermates. Filtering Log2 fold change ≥ 0.59 (up-regulated in controls) or Log2 fold change ≤ ^-^0.59 (down-regulated in controls) and adjusted *P*-value ≤ 0.1 are depicted via dotted lines and filtered populations are displayed as down-regulation in the controls (as compared to the knockout [blue]) and up-regulation in controls (as compared to knockout [red]). Top 25 genes are labeled with gene abbreviations.


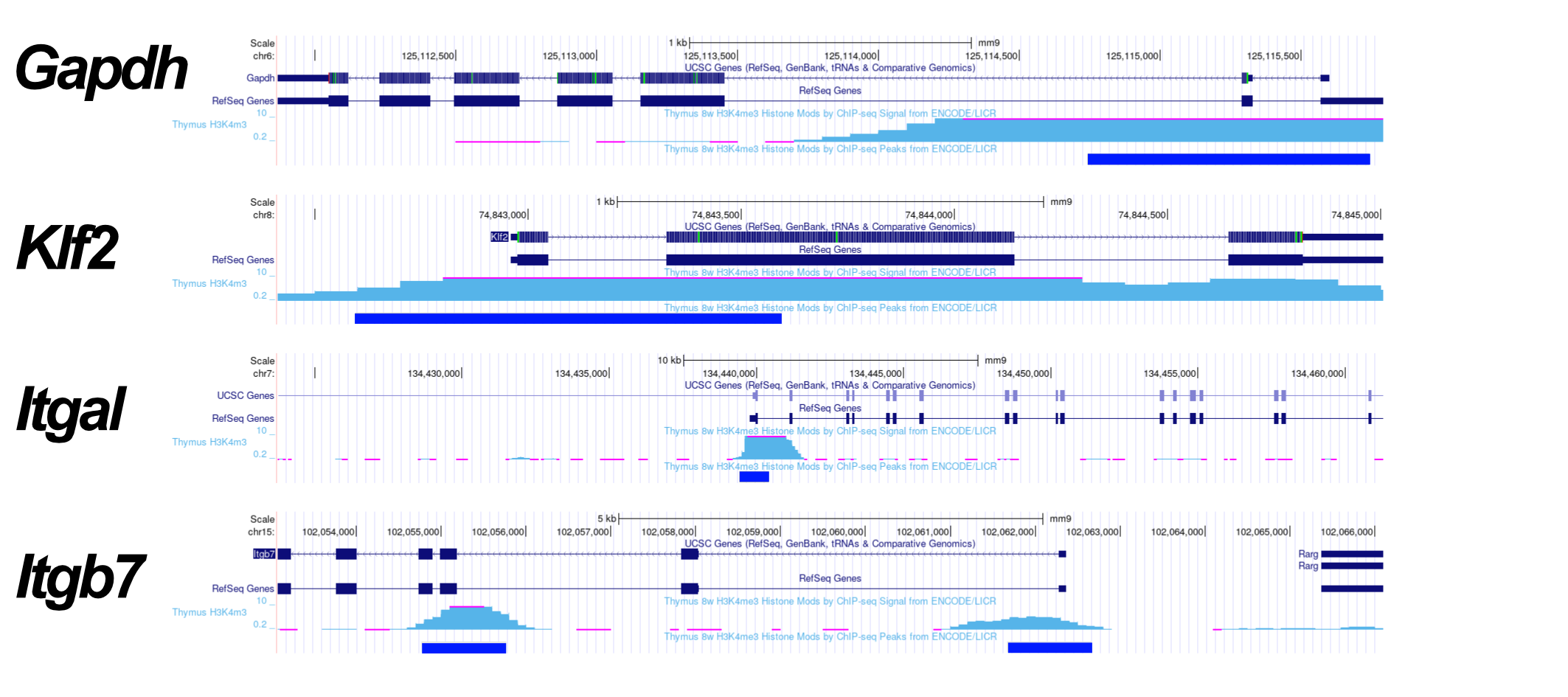

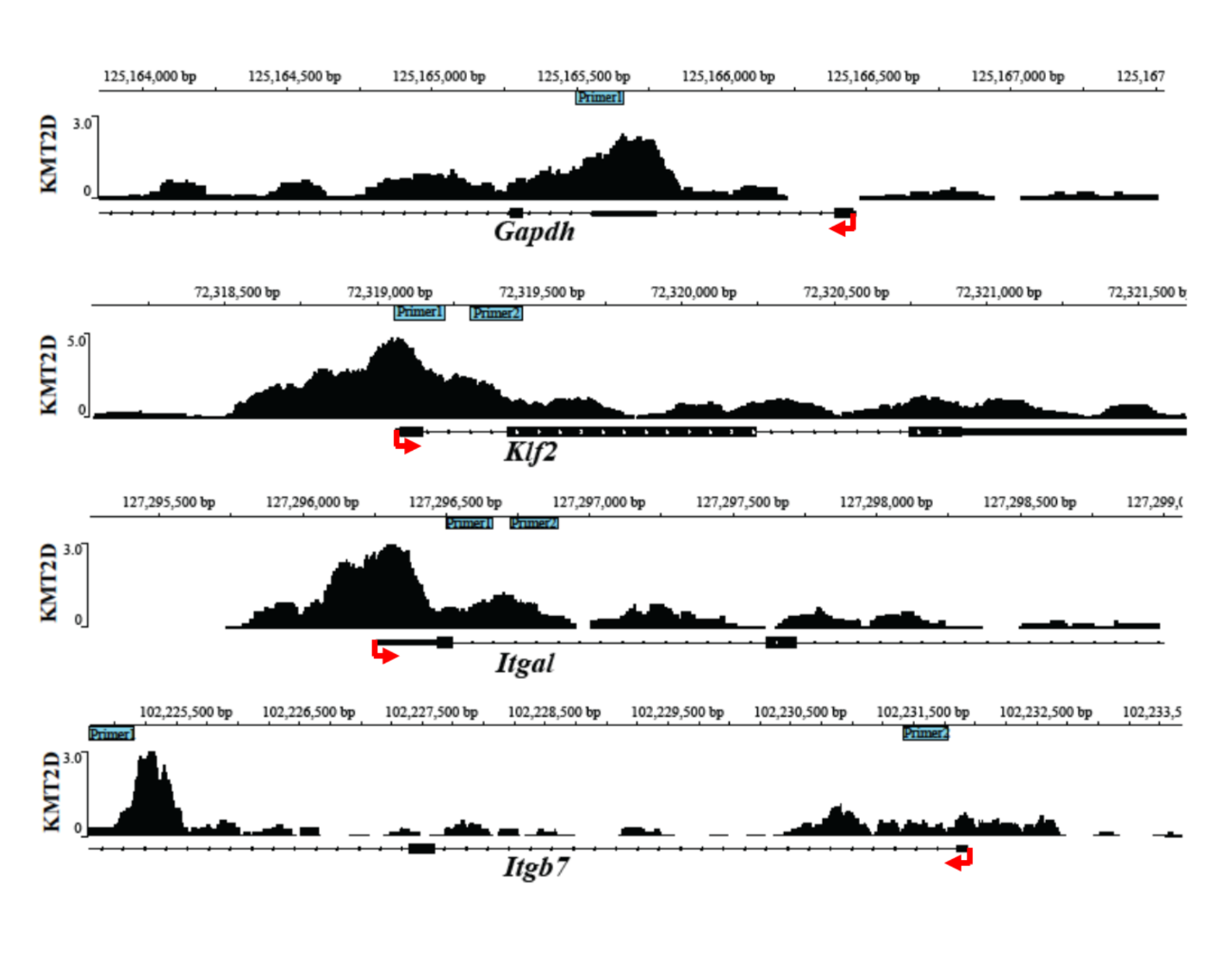


**Supplementary Figure 4. Primer location selection based on known thymic sites of enriched H3K4me3 in important KMT2D modulated genes. (Upper panel)** H3K4me3 bulk thymocyte track regions demonstrated in order: UCSC gene track (dark blue), RefSeq gene track (dark blue), H3K4me3 bulk 8 wk thymocyte peaks (light blue/pink), called peaks [bars; bright blue]. Called peaks regions were used for primer design locations for **Figure 3** ChIP-PCR. (**Bottom panel**). Visualization of the primer location by Integrative Genomics Viewer (IGV) browser image in relative range of chromatin immunoprecipitation (ChIP) binding of KMT2D in control naïve peripheral CD4^+^ T cells at *cis*-regulatory regions roughly -1 kb to + 2.5 / 5 kb around the transcription start site (TSS) region of the *Kmt2d* KO down-regulated gene (*Itgb7*, *Itgal*, and *Klf2*) or a housekeeping gene control (*Gapdh*) from GEO accession number: GSE69162. Red arrows note the TSS direction.

**Supplementary Figure 5. Observed thymic aberrations in *Kmt2d*-deficient models.** (**A**) Image of dissected *Lck*-Cre^Mar^ *Kmt2d*-KO thymi (displaying hypertrophy) compared to littermate controls. (**B**) Cleavage of CD44^+^ is required for SP to egress. Accumulation of CD44^+^TCRβ^+^ SP population (as a percent of CD4^+^SP [open] or CD8^+^SP [closed]) in *Kmt2d*-KO (*CD4-*Cre; *Kmt2d*^-SET-fl/fl^ [Cre^-^ black/Cre^+^ red triangles]) and *Kmt2d*-haploinsufficient (*Kmt2d^+/βgeo^* [*^+/+^* black/*^+/βgeo^* grey diamonds]) cells. (**C**/**D**) Thymic *Kmt2d*-haploinsufficient (*Kmt2d^+/βgeo^* [*^+/+^* black/*^+/βgeo^* grey diamonds]) SP maturation by flow cytometry, progressing from Mature 1 CD24^+^TCRβ^+^ (M1; left) to CD24^-^TCRβ^+^ Mature 2 (M2; right) as a percent of total thymocytes (**C**) or by M2-SP/ M1-SP ratio using values from % of total thymocytes values (**D**). Each dataset displays black lines, which represent mean ± SEM. Significance labeled on graphs were determined using a parametric, unpaired, Welch’s corrected *t-test* with *P*-values noted by stars based on *P*-value > 0.05 (ns), *P*-value < 0.05 (*) and *P*-value < 0.0001 (****).

**
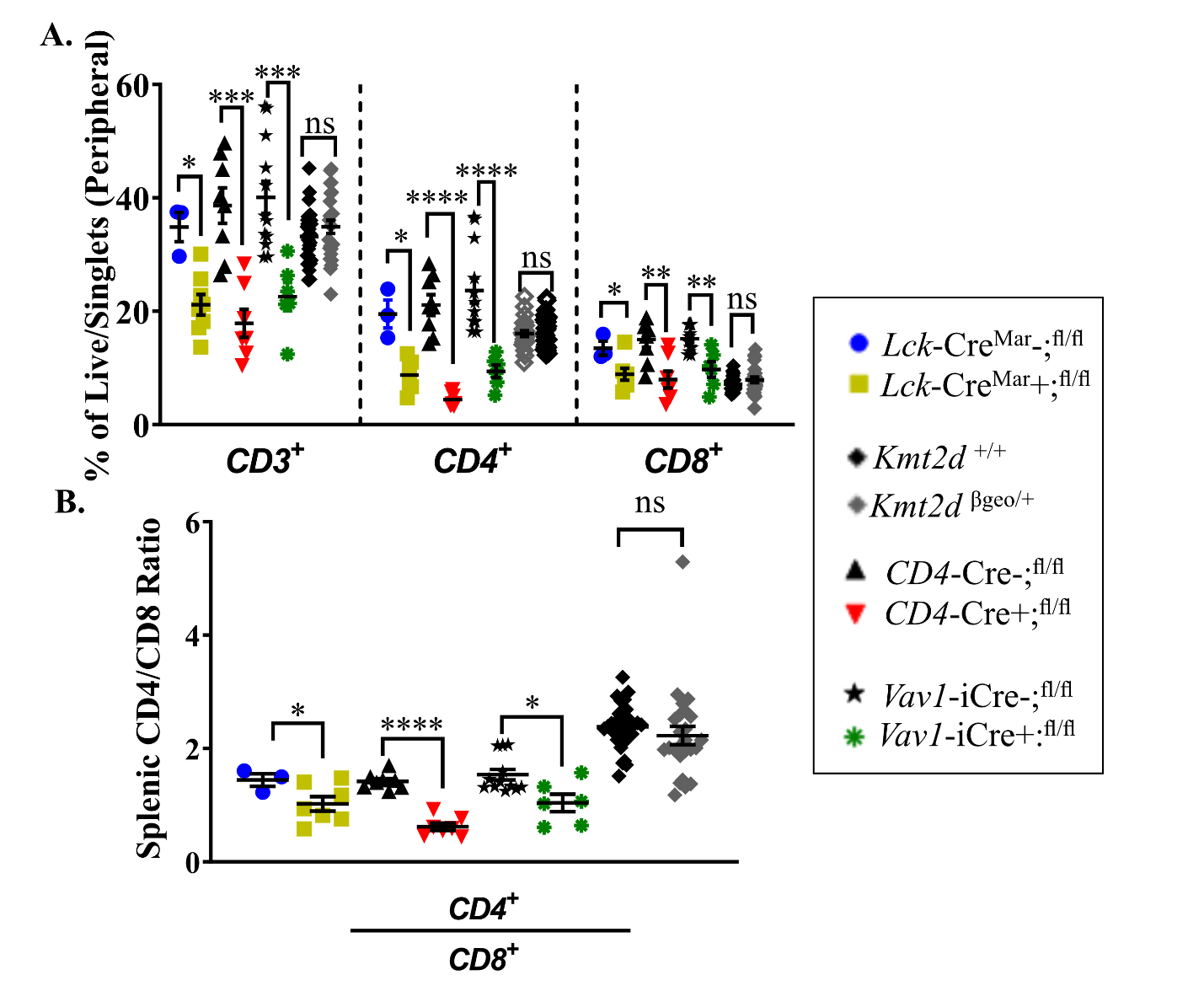
**

**Supplementary Figure 6.** ***Kmt2d*-deficient** **peripheral T cells demonstrate decreased total T cells in live/single cells of spleen and altered CD4^+^/CD8^+^ ratios.** (**A**) Assessment of splenic T cell subpopulations as a percent of total live/singlets cells. (**B**) Ratio of splenic CD4^+^/CD8^+^ in all mouse strains. Significance levels labeled on graphs were determined using a parametric, unpaired, Welch’s corrected *t-test* with *P*-values noted by asterisks: *P*-value > 0.05 (ns), *P*-value < 0.05 (*), *P*-value < 0.01 (**), *P*-value < 0.001 (***), and *P*-value < 0.0001 (****).

**
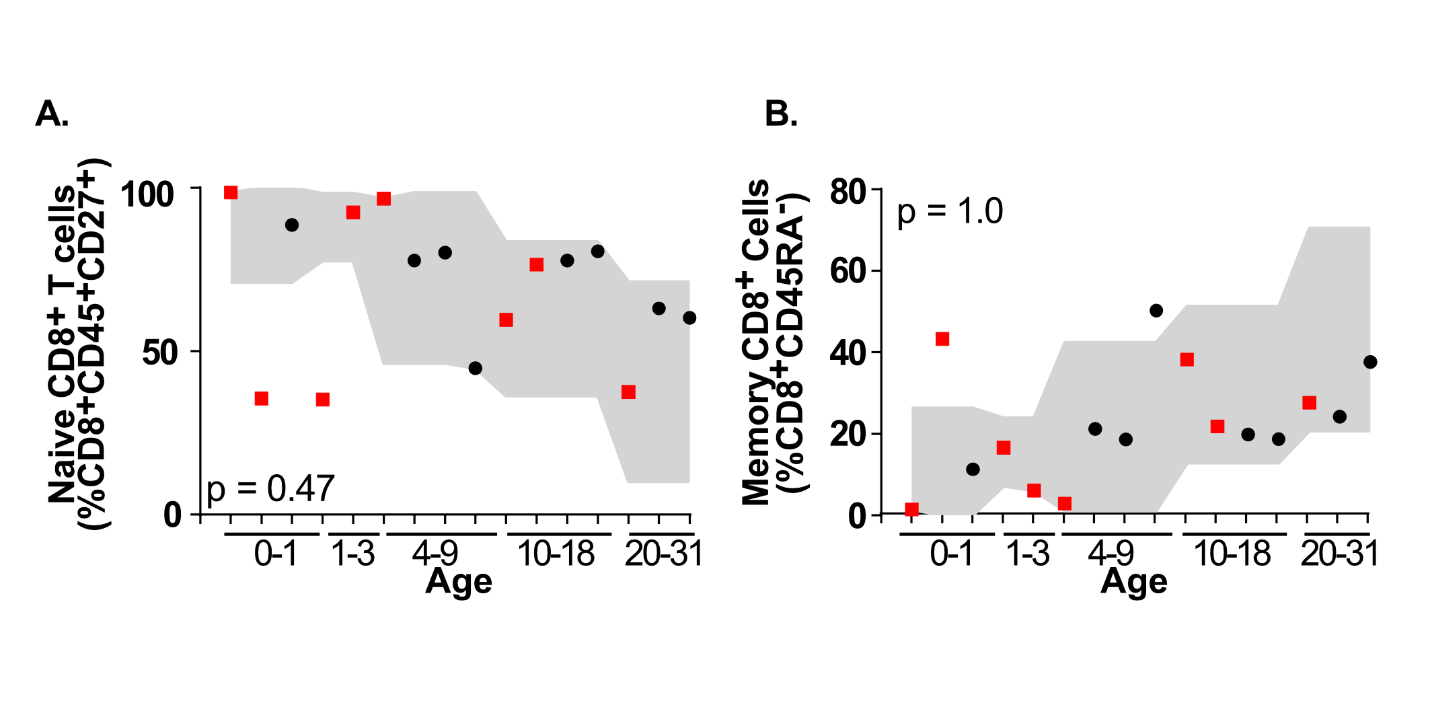
Supplementary Figure 7. Naïve and memory CD8^+^ T from individuals with KS1 cells fall within the standard range.** Data from clinical flow cytometry. Naïve CD8^+^ T cells (CD8^+^CD45RA^+^CD27^+^; **A**) and Memory CD8^+^ T cells (CD8^+^CD45RA^-^; **B**) as a percent of CD8^+^ cells. Grey region represents reference population range [2.5% - 97.5%] for the healthy age-matched individuals. Significance (shown as *P*-values listed on chart) was determined using the binomial test, with the null hypothesis being that individuals with KS1 have a same probability of falling outside the reference range equal to 5%, that is, the same as healthy age-matched individuals. The resulting *P*-values were subsequently adjusted for multiple testing with the Bonferroni method. Cardiac surgery is required by many KS1 individuals and the procedure can potentially remove and/or damage the thymus. Depicted surgery status of the individuals (cardiac surgery, red square; non-surgery, black circle) is does not significantly influence data.
